# LSM14B is an Oocyte‐Specific RNA‐Binding Protein Indispensable for Maternal mRNA Metabolism and Oocyte Development in Mice

**DOI:** 10.1002/advs.202300043

**Published:** 2023-04-21

**Authors:** Hui Li, Hailian Zhao, Chunhui Yang, Ruibao Su, Min Long, Jinliang Liu, Lanying Shi, Yuanchao Xue, You‐Qiang Su

**Affiliations:** ^1^ Shandong Provincial Key Laboratory of Animal Cells and Developmental Biology School of Life Sciences Shandong University Qingdao 266237 P. R. China; ^2^ State Key Laboratory of Reproductive Medicine Nanjing Medical University Nanjing 211126 P. R. China; ^3^ Institute of Biophysics Chinese Academy of Sciences Beijing 100101 P. R. China; ^4^ Collaborative Innovation Center of Genetics and Development Fudan University Shanghai 200433 P. R. China

**Keywords:** female infertility, LSM14B, mRNA metabolism, oocytes, RNA‐binding proteins (RBPs), ribonucleoprotein (RNP) complexes

## Abstract

Mammalian oogenesis features reliance on the mRNAs produced and stored during early growth phase. These are essential for producing an oocyte competent to undergo meiotic maturation and embryogenesis later when oocytes are transcriptionally silent. The fate of maternal mRNAs hence ensures the success of oogenesis and the quality of the resulting eggs. Nevertheless, how the fate of maternal mRNAs is determined remains largely elusive. RNA‐binding proteins (RBPs) are crucial regulators of oogenesis, yet the identity of the full complement of RBPs expressed in oocytes is unknown. Here, a global view of oocyte‐expressed RBPs is presented: mRNA‐interactome capture identifies 1396 RBPs in mouse oocytes. An analysis of one of these RBPs, LSM family member 14 (LSM14B), demonstrates that this RBP is specific to oocytes and associated with many networks essential for oogenesis. Deletion of *Lsm14b* results in female‐specific infertility and a phenotype characterized by oocytes incompetent to complete meiosis and early embryogenesis. LSM14B serves as an interaction hub for proteins and mRNAs throughout oocyte development and regulates translation of a subset of its bound mRNAs. Therefore, RNP complexes tethered by LSM14B are found exclusively in oocytes and are essential for the control of maternal mRNA fate and oocyte development.

## Graphical abstract

Through capturing the mouse oocyte RBPome, LSM14B is identified to be an oocyte‐specific RBP that is indispensable for the control of oocyte maturation and the fate of maternal mRNAs. The mRNA‐ and protein‐interaction partners of LSM14B are revealed, which indicate that LSM14B forms dynamic and versatile RNP complexes with proteins and mRNAs that are crucial for mammalian oocyte development.

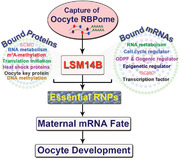

## 1 Introduction

A unique mode of regulation of gene expression exists in oocytes of most animal species that ensures the establishment of an oocyte‐specific gene expression program to drive oogenesis.^[^ ^1^ ^]^ In mammals, oocyte transcription occurs in the growth phase and gradually ceases as the oocyte approaches its final growth phase and acquires competence to resume meiosis. This transcriptional silent state lasts for the entire process of oocyte meiotic maturation, and new transcription event will not take place until after fertilization when zygotic genome activation (ZGA) initiates in the early‐stage embryos.^[^ ^2^ ^]^ Maternal mRNAs accumulated in the growing oocytes are vital for supporting oocyte maturation, fertilization and early‐stage embryo development, and regulation of maternal mRNA metabolism is essential for controlling gene expression in oocytes.^[^ ^3^ ^]^

Oocyte mRNAs are mostly stable before oocyte maturation, a condition that enables storage of maternal messages for later use.^[^ ^1^ ^]^ However, the fate of stored maternal mRNAs changes when oocytes reach the fully‐grown stage and undergo meiotic maturation. Some mRNAs are translated to support oocyte maturation, whereas many others are degraded in preparation for establishing the embryo‐specific developmental program.^[^ ^1^, ^3^, ^4^ ^]^ Specific degradation of certain mRNAs also occurs in the growing oocytes, which is important for oocyte acquisition of meiotic and developmental competence, and for maintaining genome integrity.^[^ ^5^ ^]^ Therefore, the fate of maternal mRNAs ensures the success of oogenesis and the quality of the resulting eggs. Nonetheless, how the fate of maternal mRNAs is determined remains elusive.

RNA‐binding proteins (RBPs) are major participants in the regulation of oocyte mRNA metabolism,^[^ ^6^ ^]^ however, the identity and function of the full complement of RBPs expressed in oocytes remains deficient. Here, after capturing and characterizing the RBPome of mouse oocytes, we put forward a global view of the oocyte‐expressed RBPs and discovered the role of LSM family member 14B (LSM14B)‐directed RNA‐protein interaction networks associated with the regulation of maternal mRNA metabolism and oogenesis.

## 2 Results

### 2.1 Capture of the Mouse Oocyte RBPome

**Figure 1:**
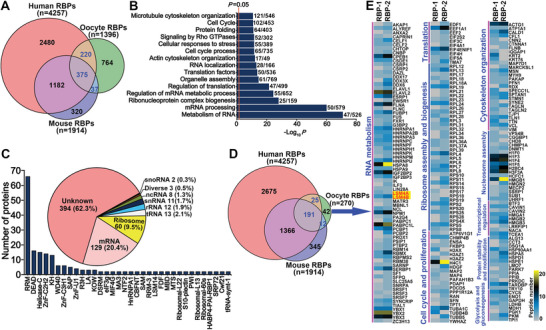
Identification and characterization of RBPs expressed in fully‐grown mouse oocytes. A) Venn diagram comparing the oocyte RBPs identified here with the human and mouse RBP datasets extracted from the EMBL RBPbase. B) Bar graph illustrating the pathways associated with the “confirmed oocyte RBPs” (defined by the presence in the RBPbase). C) The RNA binding properties and RBD compositions of the “confirmed oocyte RBPs.” The pie chart demonstrates the number and proportion of each type of RBP as categorized by the properties of their binding targets. The bar graph shows the number of RBPs with a specified RBD. D) Venn diagram comparing the refined list of mouse oocyte RBPs (defined by the MS‐peptide number ≥2) with the human and mouse RBP datasets. E) Heatmaps illustrating the peptide abundance and functional properties of the refined oocyte RBPs that are also present in the RBPbase.

To gain a global view of the mRNA interacting RBPs in oocytes, we performed mRNA interactome capture (RIC) analysis using 50 000 fully‐grown oocytes (FGOs) (equivalent to ≈10^8^ HeLa cells in the amount of total proteins) collected from normal wild type prepubertal mice, as previously reported by Hentze Group.^[^ ^7^ ^]^ This approach combines UV cross‐linking and oligo(dT) capture to pull down mRNA‐bound RBPs in live oocytes for subsequent quantitative mass spectrometry (MS) (Figure S1A,B, Supporting Information). This analysis identified 1149 and 549 putative RBPs in the first and the second biological replicate, respectively, which gave rise to 1396 total putative RBPs in two independent experiments (Dataset S1, Supporting Information). There were 302 proteins present in both replicates, the intensity (iBAQ) of which correlated well between replicates (*R* = 0.86, *p* < 0.0001) (Figure S1C,D, Supporting Information). By comparing the 1396 putative mouse oocyte RBPs identified here with publicly available human and mouse RBP datasets extracted from the EMBL RBPbase (https://rbpbase.shiny.embl.de/), we defined 632 overlapping proteins as “confirmed RBPs,” while the remaining 764 proteins were designated as “unknown RBPs” (**Figure** **1**A; Dataset S1, Supporting Information). Gene enrichment analysis suggested that the former set was mainly involved in processes related to “mRNA metabolism and processing” (Figure 1B). In contrast, the latter set was associated with diverse processes unrelated to RNA biology (Figure S1C, Supporting Information).

Further manual curation of the 632 “confirmed RBPs” revealed a subset of 238 (37.7%) RBPs known to bind RNA, while another subset of 394 (62.3%) RBPs were not yet annotated for their role in RNA binding (Figure 1C; Dataset S1, Supporting Information). Among well‐annotated RBPs, the vast majority were ribosomal or mRNA‐binding proteins (Figure 1C; Dataset S1, Supporting Information). Moreover, 225 of the “confirmed RBPs” contained at least one defined RNA‐binding domain (RBD) in their protein structure, with the most prevalent RBDs including RRM, DEAD, Helicase‐C, ZnF‐C2H2, and KH (Figure 1C; Dataset S1, Supporting Information).

The list of oocyte RBPs was defined further by including only proteins with a detected MS‐peptide number ≥2, resulting in a smaller set of 270 proteins (188 in the first biological replicate, 255 in the second biological replicate, and 143 in both replicates) that comprised the oocyte RBPome, 228 of which were found in the EMBL RBPbase (Figure 1D; Figure S1C and Dataset S1, Supporting Information). Two‐thirds of the 228 known RBPs were predicted to participate in processes related to “RNA metabolism” and “cell cycle,” while the others were involved in “regulation of cytoskeleton organization,” “nucleosome assembly/transcription,” “protein stability/modification, and glycolysis/gluconeogenesis” (Figure 1E). Notably, this protein list included both RBPs with well‐established, essential functions in oogenesis (e.g., CPEB1,^[^ ^8^ ^]^ DAZL,^[^ ^9^ ^]^ and YBX2 (MSY2)^[^ ^10^ ^]^), as well as others with currently undefined roles in mammalian oogenesis (e.g., LSM14A/B^[^ ^11^ ^]^ and RBPMS2^[^ ^12^ ^]^). This mouse oocyte RBPome provides a rich resource for exploring the fate‐determination mechanisms for maternal mRNAs.

### 2.2 Defining LSM14B as an Oocyte‐Specific RBP Essential for Mouse Oogenesis

**Figure 2:**
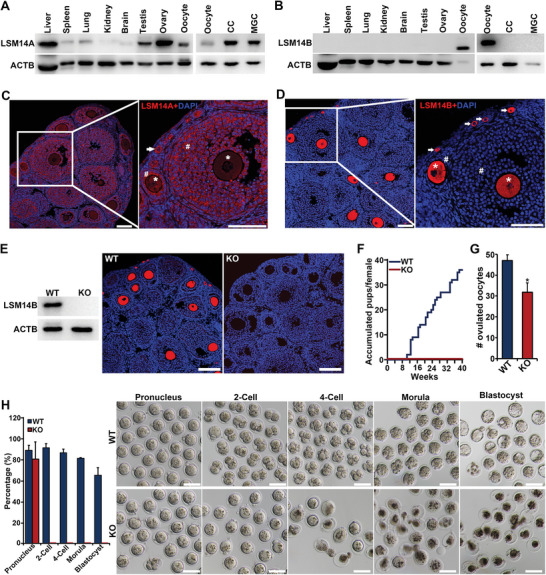
Oocyte‐specific expression and function of LSM14B in mice. A,B) Western blot (WB) analysis of the expression of LSM14A and B in various mouse tissues, and the cells within the large antral follicle, that is, the oocyte, cumulus cell (CC), and mural granulosa cell (MGC) (*n* = 2). In Panel A, 400 fully‐grown oocytes (FGOs) were loaded for comparison with various mouse tissues, and 50 FGOs were loaded for comparison with CC and MGC. In Panel B, 100 FGOs were loaded for comparison with various mouse tissues, and 50 FGOs were loaded for comparison with CC and MGC. C,D) Immunofluorescence (IF) staining of LSM14A and B in the ovaries of 21 day old mice (*n* = 2). The magnified views of the boxed areas are shown in the right panels, with the arrow pointing to the non‐growing oocyte in the primordial follicles, * indicating the growing and fully‐grown oocytes, and # showing granulosa cells. LSM14A and B are stained in red, and DNA is counterstained in blue. Scale bars represent 100 µm. E) WB and IF staining showing the complete deletion of LSM14B protein in *Lsm14b*‐KO oocytes. LSM14B is stained in red, and DNA is counterstained in blue (*n* = 3). F) Fertility test of the wild type (WT, *n* = 4)‐ and *Lsm14b*‐KO (KO, *n* = 6) female mice. G) Oocyte count after superovulation of the 24 day old WT‐ and *Lsm14b*‐KO female mice. Data are the mean ± s.e.m (*n* = 11 and 7 in the WTs and KOs, respectively). *p < 0.05, compared with the KOs by student *t*‐test. H) The rate (left bar graph) and representative micrograph (right panel) of pronucleus, 2‐cell, 4‐cell, morula, and blastocyst embryos formed by ovulated WT‐ and *Lsm14b*‐KO oocytes after in vitro fertilization with WT‐ normal sperm (*n* = 2). Scale bars represent 100 µm.

LSM14A and LSM14B are two vertebrate paralogs of the highly conserved LSM14 protein family implicated in the regulation of maternal mRNA metabolism and oogenesis in various species.^[^ ^11^, ^13^ ^]^ They were, therefore, selected to further investigate the potential participation of newly identified oocyte RBPs in the regulation of the fate of maternal mRNA. Their expression in mouse oocytes was first verified by Western blot (WB) and immunofluorescence (IF) analyses (**Figure** **2**A–D). LSM14A was detectable in most of the mouse tissues and was generally restricted to cumulus and mural granulosa cells of the large antral follicles within the ovary (Figure 2A,C). LSM14A was barely detectable in FGOs although prominent expression was observed in oocytes of primordial follicles (Figure 2A,C). In contrast, LSM14B was exclusively expressed in the oocytes at all follicle stages, and not in granulosa cells or any of the other tissues (Figure 2B,D). These distinct expression patterns between the two LSM14 paralogs in mice suggested that LSM14B might function as an oocyte‐specific RBP in mammals.

**Figure 3:**
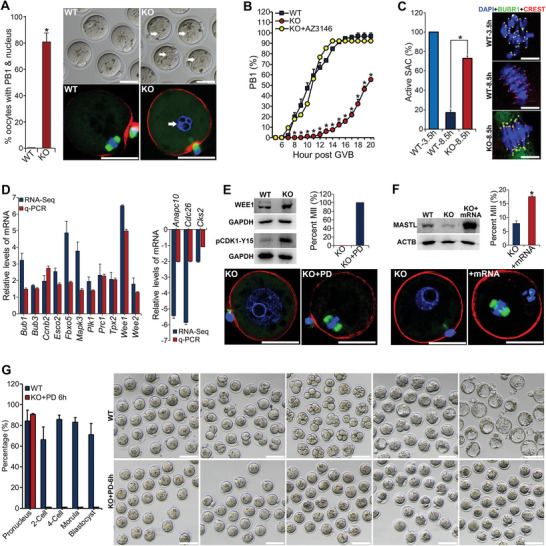
Compromised progression of meiosis to metaphase II in *Lsm14b*‐KO oocytes. A) The oocytes ovulated by *Lsm14b*‐KO mice have a visible first polar body (PB1) and pronucleus (PN). Data are the mean ± s.e.m (*n* = 3). *p < 0.05, compared with the KOs by student *t*‐test. Representative microphotographs of the WT‐ and *Lsm14b*‐KO‐ ovulated oocytes are shown on the right, with IF staining of the oocyte spindle (green), chromosome (blue) and F‐actin (red) indicated at the bottom. Arrows indicate the PN. Scale bar = 50 µm. B) Kinetics of the emission of PB1 in WT‐ and *Lsm14b*‐KO‐oocytes, and the *Lsm14b*‐KO‐oocytes treated with the MPS1 inhibitor (AZ3146) during IVM. C) Defective inactivation of SAC in *Lsm14b*‐KO oocytes. The bar graph shows the quantification of the percentage of oocytes with BUBR1 positively stained at the kinetochores (active spindle assembly checkpoint) after IVM for 3.5 or 8.5 h. Data are the mean ± s.e.m (*n* = 3). *p < 0.05 by Student's *t*‐test. The right micrographs demonstrate the IF staining of BUBR1 (green), CREST (red), and DNA (blue) in the oocytes after IVM for 3.5 or 8.5 h. Scale bar = 5 µm. D) Changes of the expression of meiotic progression‐related genes in *Lsm14b*‐KO‐oocytes as detected by RNA‐Seq and q‐PCR. The levels of the WT groups were normalized to 1 in both the RNA‐Seq and qRT‐PCR analyses, which were not shown in the figure. E) Upregulation of WEE1 and pCDK1‐Y15 (inactive CDK1) protein in the GV‐stage fully‐grown oocytes (FGOs) of the *Lsm14b*‐KO‐mice (WB gel graphs), and rescue of the PN‐arrest phenotype in the ovulated *Lsm14b*‐KO‐oocytes that have a visible PN by treatment with WEE kinase inhibitor, PD16685 (the bar graph and microphotographs). G) Downregulation of MASTL protein in the GV‐stage FGOs of *Lsm14b*‐KO‐mice (WB gel graphs), and partial rescue of the PN‐arrest phenotype in *Lsm14b*‐KO‐oocytes by microinjection of the GV‐stage FGOs with *Mastl* mRNA followed by in vitro maturation (the bar graph and microphotographs). In E,F), spindles, chromosomes, and F‐actin are stained in green, blue and red, respectively, Scale bar = 50 µm. G) Compromised developmental competence in the *Lsm14b*‐KO‐oocytes. The ovulated *Lsm14b*‐KO‐oocytes that have a visible PN were rescued to MII by treatment with WEE kinase inhibitor, PD16685, for 6 h, and then fertilized in vitro with normal sperms isolated from WT‐mice. The rate and representative micrograph of pronucleus, 2‐cell, 4‐cell, morula, and blastocyst embryos was shown in the left (bar graph) and the right panels, respectively (*n* = 2). Scale bars represent 100 µm.

The possible role of LSM14B in oogenesis was investigated using mice carrying a targeted knockout allele of *Lsm14b* (*Lsm14b* ^tm1a(KOMP)Mbp^) induced by reporter‐tagged insertion. In *Lsm14b* ^tm1a(KOMP)Mbp^‐homozygous females, LSM14B protein expression in oocytes was undetected (Figure 2E), verifying that *Lsm14b* ^tm1a(KOMP)Mbp^ (hereafter referred to as *Lsm14b*‐KO) is a null allele. The *Lsm14b*‐KO male mice showed normal fertility and were used for colony maintenance. However, *Lsm14b*‐KO female mice were infertile (Figure 2F), and ovulated slightly fewer eggs than the wild types (WTs) (Figure 2G). When these mutant eggs were fertilized by WT‐sperm in vitro, neither cleavage nor blastocyst formation occurred, instead they gradually degenerated in culture (Figure 2H). Unlike the ovulated WT‐eggs that arrested at metaphase II (MII), the majority (80%) of the *Lsm14b*‐KO ovulated eggs arrested at interphase, with a prominent pronucleus (PN)‐like internal structure (**Figure** **3**A). Thus, infertility in *Lsm14b*‐KO females was caused, at least in part, by failure of oocytes to progress to metaphase II. Therefore, LSM14B is required for oocyte meiotic progression and female fertility in mice.

To characterize the full extent of defects in meiotic progression in *Lsm14b*‐KO oocytes, oocytes were injected with mRNAs encoding green fluorescent protein (GFP)‐tagged tubulin and mCherry‐tagged histone. In addition to confirming PN formation, in vitro maturation (IVM) and live‐cell imaging revealed that MI‐to‐anaphase I (AI) transition and the first polar body emission (PBE) were both significantly delayed (3–5 h) in *Lsm14b*‐KO oocytes (Figure 3B; Figure S2A,B, and Movies S1 and S2, Supporting Information). In accordance with the delayed onset of AI, a large proportion (73.03%) of *Lsm14b*‐KO MI‐oocytes were stained positively for BUB1B (BUBR1) expression at their kinetochores, indicating prolonged activation of the spindle assembly checkpoint (SAC) (Figure 3C). Chemical inhibition of the upstream TTK (MPS1) kinases by AZ3146 treatment to pass the SAC led to complete rescue of the prolonged MI and delayed PBE defects in *Lsm14b*‐KO oocytes (Figure 3B). Despite prolonged SAC activation, no differences were observed between WT‐ and Lsm14b‐KO oocytes in either the timing of meiotic progression to MI, the MI‐spindle assembly and chromosome alignment, or microtubule attachment to kinetochores (Figure S2A,B,D, and Movies S1 and S2, Supporting Information). These results led us to hypothesize that the prolonged MI was related to a failure to initiate anaphase, rather than defects leading to SAC activation.

To test this possibility, the activity of the anaphase‐promoting complex (APC) was estimated by injecting WT‐ and *Lsm14b*‐KO oocytes with mRNAs encoding Venus‐tagged CCNB1 (Cyclin B1) and mCherry‐tagged PTTG1 (Securin) and monitored dynamic changes in these proteins in oocytes during IVM via live‐cell imaging (Figure S3, Supporting Information). While CCNB1‐Venus abruptly declined in WT‐oocytes at ≈13 h post IVM, immediately prior to AI onset, the decrease in CCNB1‐Venus was considerably less and significantly delayed in *Lsm14b*‐KO oocytes (Figure S3, Supporting Information). Moreover, in the absence of *Lsm14b*, CCNB1‐Venus levels were not partially restored after MI‐to‐AI transition as observed in WT oocytes (Figure S3, Supporting Information), which could consequently lead to low levels of MPF and meiotic entry into interphase. We also observed similar defects in PTTG1‐mCherry dynamics in the *Lsm14b*‐KO oocytes (Figure S3, Supporting Information). Collectively, these data indicated that the prolonged MI and failed meiotic progression to MII phenotypes of *Lsm14b*‐KO oocyte were most likely caused by delayed activation of the APC at the end of MI and an inability to reactivate maturation‐promoting factor (MPF) after MI‐to‐AI transition.

### 2.3 Mis‐Expression of Genes Crucial for Meiotic Progression in *Lsm14b*‐KO Oocytes

To gain more mechanistic insights on how *Lsm14b*‐KO caused oocyte meiotic defects, transcriptome‐ and proteome‐wide changes in gene expression in *Lsm14b*‐KO oocytes were investigated. Transcriptomic analysis identified 2285 differentially expressed genes (DEGs; 970 up, 1315 down) between WT and *Lsm14b*‐KO immature germinal vesicle (GV)‐stage FGOs (Figure S4A, Supporting Information). These were enriched in distinct categories of biological pathways (Figure S6B,C, and Dataset S2, Supporting Information). Upregulation of *Bub1*, *Bub3*, *Ccnb2*, *Esco2*, *Fbxo5* (*Emi1*), *Mapk3* (*Erk1*), *Plk1*, and *Tpx2*, and the downregulation of *Anapc10*, *Cdc26* (*Anapc12*), and *Cks2* in the *Lsm14b*‐KO oocytes was found and validated (Figure 3D). Since these genes are reportedly crucial for the control of oocyte meiotic progression,^[^ ^14^ ^]^ their mis‐expression in oocytes could account for the phenotypes of prolonged MI and delayed MI‐to‐AI transition, respectively. In addition, *Wee1* and *Wee2* mRNAs were also upregulated in *Lsm14b*‐KO oocytes, as was WEE1 protein (Figure 3D,E). As negative regulators of CDK1,^[^ ^15^ ^]^ WEE upregulation could maintain low levels of MPF activity following MI‐to‐AI transition, thereby preventing oocyte entry to MII. Indeed, pCDK1‐Y15 (i.e., inactive CDK1) levels were elevated in *Lsm14b*‐KO oocytes, and treatment with WEE kinase inhibitor, PD166285, effectively induced MII in *Lsm14b*‐KO oocytes (Figure 3E).

Proteomic analysis identified 326 differentially abundant proteins between *Lsm14b*‐KO and WT oocytes, which were notably enriched in “membrane trafficking and protein transport,” “cell cycle,” and “‘RNA/amino acid/carbon metabolism” pathways (Dataset S3 and Figure S5A–C, Supporting Information). Comparison with the above DEGs identified 84 (23 down, 28 up) protein/mRNA pairs common to both datasets (Figure S5D–F, Supporting Information). However, no change in expression was detected for mRNAs corresponding to a large proportion (>84%) of the differential proteins, indicating the involvement of posttranscriptional regulation. Among these discrepant mRNA/protein pairs, proteomic and WB analysis showed that MASTL protein was almost undetectable in *Lsm14b*‐KO oocytes but abundant in WT (Figure 3F **;** Dataset S3, Supporting Information). Since *Mastl* deletion reportedly induces the same phenotype as *Lsm14b*‐KO in oocytes,^[^ ^16^ ^]^ whether downregulation of MASTL protein contributed to the meiotic defects in *Lsm14b*‐KO oocytes was investigated. Microinjection with *Mastl*‐mRNA to increase MASTL protein levels in *Lsm14b*‐KO oocytes led to 50% higher numbers of MII‐oocytes (Figure 3F), indicating that downregulation of MASTL could contribute, at least in part, to meiotic failure in *Lsm14b*‐KO oocytes.

Interestingly, the *Lsm14b*‐KO MII‐stage oocytes rescued by the WEE kinase inhibitor, PD166285, were able to be fertilized in vitro by the WT‐sperm, and formed pronuclei (Figure 3G). However, these pronucleus‐stage embryos derived from the *Lsm14b*‐KO oocytes failed to cleave and develop further (Figure 3G), thus implying that LSM14B is also crucial for oocyte acquisition of full developmental competence.

### 2.4 Identification of LSM14B‐Bound mRNAs in Oocytes by LACE‐Seq

**Figure 4:**
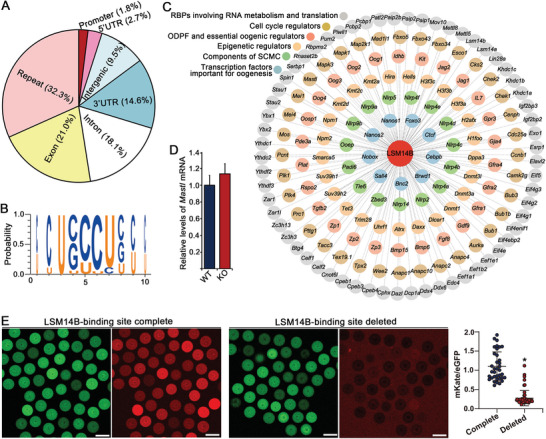
Characterization of LSM14B‐bound RNAs identified in oocytes by CLIP and LACE‐Seq. A) Pie chart depicting the genomic distribution of LSM14B binding sites. B) Sequence logo representation of the LSM14B‐binding consensus calculated from the top‐20 enriched hexamers. C) Network illustration of the specific subset of LSM14B‐bound mRNAs typified by those encoding known “star factors” of oogenesis. D) qRT‐PCR comparison of *Mastl* mRNA levels in WT‐ and *Lsm14b*‐KO FGOs. Data are the mean ± s.e.m (*n* = 3). No significant difference between the WTs and KOs by Student's *t*‐test. E) UTR‐reporter assay to evaluate the effect of LSM14B binding to 3′UTR on Mastl mRNA translation. “Complete” indicates that the 3′UTR sequence of *Mastl* mRNA is intact, while the “Deleted” refers to the binding site for LSM14B is deleted from the 3′UTR sequence of Mastl mRNA. Scale bar = 100 µm.

The linear amplification of complementary DNA ends and sequencing (LACE‐Seq) method developed by us recently was used to identify LSM14B‐bound mRNAs in mouse oocytes at single‐nucleotide resolution.^[^ ^17^ ^]^ In total, 78 741 binding peaks were detected genome‐wide, mapping to 9253 mRNAs that were enriched in a variety of processes and pathways (Dataset S4 and Figure S6A, Supporting Information). Fifteen percent of the binding sites were within the 3′UTR of LSM14B‐bound mRNAs, with CCUCUC serving as the most enriched hexamer (**Figure** **4**A,B). Manual curation of these LSM14B‐bound mRNAs further identified a subset of transcripts typified by those encoding “star factors,” proteins well‐established to play essential roles in oogenesis (Figure 4C; Table S2, Supporting Information), including 1) RBPs involved in RNA metabolism and translation crucial for oocyte nuclear and cytoplasmic maturation, for example, PATL2,^[^ ^18^ ^]^ YBX2,^[^ ^10^ ^]^ and ZAR1;^[^ ^19^ ^]^ 2) cell cycle‐regulatory factors essential for oocyte meiotic progression, for example, CKS2,^[^ ^14g^ ^]^ MOS,^[^ ^20^ ^]^ and WEE2;^[^ ^21^ ^]^ 3) key oocyte‐derived paracrine factors (ODPF) and oocyte and follicle developmental regulatory factors, for example, GDF9,^[^ ^22^ ^]^ GPR3,^[^ ^23^ ^]^ and PDE3A;^[^ ^24^ ^]^ 4) Epigenetic regulators essential for maintenance of oocyte genome integrity, and oocyte‐to‐embryo transition, for example, DPPA3 (Stella),^[^ ^25^ ^]^ NPM2,^[^ ^26^ ^]^ and TRIM28;^[^ ^27^ ^]^ 5) components of the oocyte‐specific subcortical maternal complex (SCMC) also essential for oocyte‐to‐embryo transition, for example, NLRP5 (MATER),^[^ ^28^ ^]^ PADI6,^[^ ^29^ ^]^ and TLE6;^[^ ^30^ ^]^ and 6) transcriptional factors essential for oocyte development, for example, BRWD1,^[^ ^31^ ^]^ FOXO3,^[^ ^32^ ^]^ and NOBOX.^[^ ^33^ ^]^ The binding of LSM14B to these “star factor” transcripts in oocytes suggests that it performs key functions in coordinating RNA‐protein interaction networks necessary for normal oocyte and follicle development.

Additionally, LACE‐Seq revealed that *Mastl* mRNAs were bound to LSM14B through three binding sites, two in the *Mastl* coding region and one in the 3′UTR (Dataset S4, Supporting Information). Since the data presented above show that MASTL protein was significantly downregulated in *Lsm14b*‐KO oocytes, we speculated that LSM14B could post‐transcriptionally regulate *Mastl* expression by either binding or stabilizing its transcripts or by promoting their translation. Supporting this possibility, steady state levels *Mastl* mRNA did not significantly differ between *Lsm14b*‐KO and WT oocytes (Figure 4D), indicating that LSM14B did not affect *Mastl* mRNA stability. To verify whether LSM14B could promote MASTL translation, live‐cell imaging of WT oocytes was used after injection with complete or LSM14B‐binding site‐deleted *Mastl*‐3′UTRs tethered downstream of an mKate fluorescence reporter (Figure S6B, Supporting Information). Co‐injection of complete mKate‐fused UTR with EGFP control transcripts in WT oocytes resulted in obvious fluorescence expression of both mKate and EGFP reporters, whereas injection with the binding site‐deleted *Mastl*‐3′UTR reporter produced nearly undetectable mKate signal, while EGFP was fully expressed (Figure 4E). Taken together, these data suggested that LSM14B can bind to *Mastl* mRNA and promotes its translation, supporting a role for LSM14B in ensuring the translation of meiosis‐related transcripts.

### 2.5 LSM14B Promotes Translation of Oogenesis‐Related mRNAs

**Figure 5:**
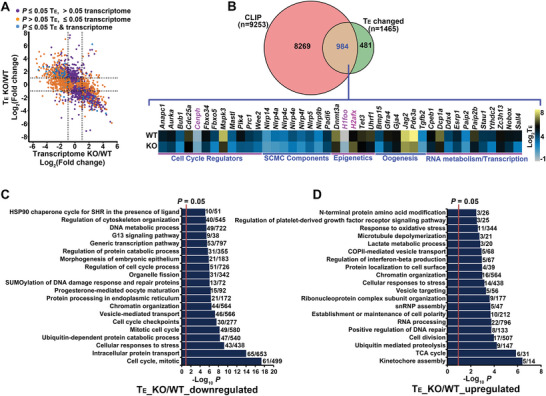
Changes of translation in *Lsm14b*‐KO‐oocytes. A) Scatterplots demonstrating the simultaneous changes of the translational efficiency (T_E_) (Y‐axis) and the steady state levels of total mRNA (transcriptome) (X‐axis) in the GV‐stage *Lsm14b*‐KO‐oocytes. B) Translational changes of the LSM14B‐bound mRNAs in the GV‐stage *Lsm14b*‐KO‐oocytes. The top Venn diagram shows the overlap of the LSM14B‐bound mRNAs (CLIP) with those having a changed T_E_, which represents the 984 LSM14B‐bound mRNAs with their translation regulated by LSM14B. The bottom heatmap shows the changes of the T_E_ of the specific group of LSM14B‐bound mRNAs that are crucial for oogenesis. C,D) Bar graphs illustrating the enriched GO/KEGG terms or canonical pathways associated with the mRNAs that are bound by LSM14B and have a decreased (C) or an increased (D) translational efficiency (T_E_) in *Lsm14b*‐KO oocytes.

A genome‐wide investigation of the effects of *Lsm14b*‐KO on mRNA translation in oocytes was conducted to obtain a broad perspective of LSM14B contributions to regulating oocyte translation. Sequencing the polysome‐loaded mRNAs in the GV‐stage FGOs followed by calculating translational efficiency (T_E_; i.e., ratio of polysome‐loaded to total transcripts) for each mRNA identified 1465 transcripts with significantly different T_E_ between *Lsm14b*‐KO and WT oocytes (Dataset S5, Supporting Information). A large proportion of T_E_‐altered transcripts included mRNAs that were not differentially expressed in RNA‐seq analysis (**Figure** **5**A). Comparison with the LACE‐seq dataset revealed that 66.3% (984) of the T_E_‐altered mRNAs were also bound by LSM14B (Figure 5B), 74.5% (733) of which had lower T_E_ under *Lsm14b*‐KO, suggesting that LSM14B promotes their translation. These mRNAs were mainly involved in “cell cycle,” and “protein processing, trafficking, and degradation” processes (Figure 5C), while the remaining 25.5% (251) had higher T_E_ in KO oocytes and were enriched in processes related to “kinetochore assembly,” “TCA cycle,” and “RNA metabolism,” etc. (Figure 5D). Further examination indicated that 42 T_E_‐altered mRNAs, 39 of which were reduced in *Lsm14b*‐KO oocytes, were previously reported to be crucial for oogenesis (Figure 5B).^[^ ^16^, ^24^, ^28^, ^29^, ^33^, ^34^ ^]^ These findings indicated that LSM14B promotes the translation of some oogenesis‐related mRNAs.

### 2.6 Interaction of LSM14B with Proteins Essential for Maternal mRNA Metabolism and Oogenesis

**Figure 6:**
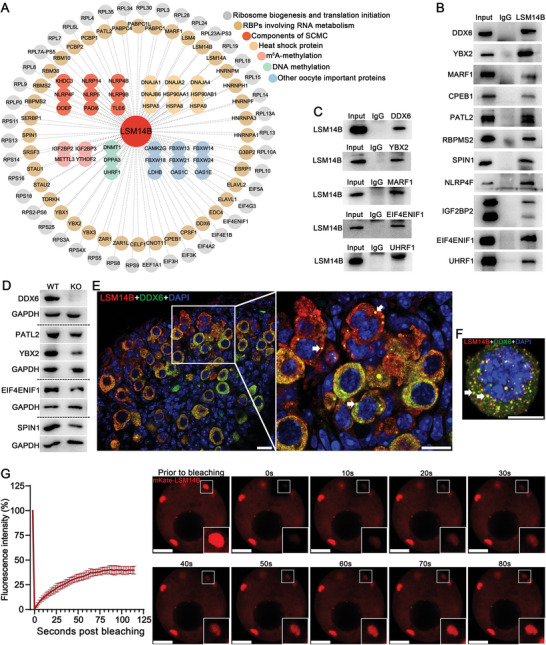
Characterization of LSM14B‐bound proteins identified in oocytes by IP‐MS. A) Network illustration of LSM14B‐bound proteins that are crucial for oogenesis. B) WB validation of binding of LSM14B to the 11 representative proteins from the IP‐MS dataset. C) WB validation of the binding of DDX6, YBX2, MARF1, EIF4ENIF1, and UHRF1 to LSM14B, respectively. D) WB analysis of the downregulation of DDX6, PATL2, YBX2, EIF4ENIF1, and SPIN1 in *Lsm14b*‐KO‐oocytes. GAPDH serves as the internal control. E,F) Colocalization of LSM14B (in red) with DDX6 (in green) in the oocytes of postnatal day‐3 ovaries (E), and the non‐growing oocytes isolated from postnatal day‐3 ovaries (F). G) FRAP analysis of the phase‐separation property of the LSM14B‐mKate (in red) formed P‐body‐like large fluorescent puncta in live early‐stage growing oocytes isolated from 6 day‐old mice after microinjection with the mRNAs for LSM14B‐mKate. Dynamic changes of the fluorescence intensity and morphology of the LSM14B‐mKate puncta before and after photo bleaching are shown in the left bar graph and the right micrographs, respectively. In E–G panels, DNA is stained in blue; arrows indicate the LSM14B and DDX6 co‐formed P‐body‐like large puncta. In G panel, insets in the bottom right corner are the magnified views of the boxed area before and after photobleaching. Scale bars in Panels E and F represent 10 µm. Scale bars in panel G indicate 5 µm.

To determine whether and which proteins might interact with LSM14B to control the fate of maternal mRNAs during oogenesis, immunoprecipitation (IP) and mass spectrometry (MS) in WT oocytes was conducted to screen for potential protein interaction partners. To ensure the robustness of IP/MS analyses, ≈8000 GV‐stage FGOs were collected in each of two independent experiments. IP/MS identified 305 putative LSM14B protein interaction partners, enriched in pathways related to “RNA metabolism,” “oocyte SCMC formation,” and “gamete generation” (Dataset S6 and Figure S7A, Supporting Information). More specifically, these proteins included 1) several ribosomal proteins and translational initiation factors/regulators (e.g., EIF4E1B and EIF4ENIF1); 2) dozens of RBPs, including several of the aforementioned “star factors” (e.g., MARF1, PATL2, YBX2, and ZAR1); 3) components of the oocyte SCMC (e.g., NLRP5, PADI6, and TLE6); 4) heat shock proteins; 5) RNA or DNA methylation regulators (e.g., IGF2BP2, YTHDF2, DNMT1, DPPA3, and UHRF1); and 6) other proteins necessary for oocyte development (e.g., CAMK2G) (**Figure** **6**A). In addition, interactions between LSM14B and some of these putative binding partners were verified by Co‐IP and WB analyses (Figure 6B,C).

Since DDX6 is a central component of processing bodies (P‐bodies) responsible for the storage or degradation of translationally repressed mRNAs,^[^ ^35^ ^]^ finding that DDX6 was interacted with LSM14B and nearly depleted in *Lsm14b*‐KO oocytes (Figure 6B–D) suggested that LSM14B might regulate mRNA metabolism with DDX6 in P‐bodies. Comparing LSM14B‐IP/MS dataset with the P‐body protein constituents previously described by Hubstenberger et al.^[^ ^36^ ^]^ revealed that 21 putative LSM14B‐binding proteins were also present in the pool of P‐body‐associated proteins (Figure S7B and Dataset S7, Supporting Information), further supporting the likelihood that LSM14B was also a component of P‐bodies in oocytes.

Prominent P‐body structures have been reported in primordial‐follicle stage oocytes of neonatal mice.^[^ ^37^ ^]^ IF co‐staining of LSM14B and DDX6 in the ovaries of postnatal day 3 mice to ascertain whether LSM14B was indeed present in the oocyte P‐body showed that LSM14 B co‐localized with DDX6 in large granular foci within the oocyte cytoplasm (Figure 6E). The granular pattern of LSM14B‐DDX6 co‐localization became increasingly prominent when the oocytes were isolated from d3‐ovarian follicles and individually stained (Figure 6F). When mouse endogenous LSM14B and DDX6 were ectopically expressed in HEK293 cells, they also co‐localized and formed granular structure resembling P‐bodies (Figure S7C, Supporting information ).

P‐bodies are well‐known ribonucleoprotein (RNP) condensates formed through phase separation.^[^ ^38^ ^]^ Fluorescence recovery after photobleaching (FRAP) in early‐stage growing oocytes isolated from 6 day‐old mouse ovaries and HEK293 cells expressing mKate‐tagged mouse LSM14B showed that the formation of LSM14B‐containing P‐body‐like structures also involves phase separation. Photobleaching specific areas of the LSM14B‐mKate aggregate abolished the fluorescent signal, but it rapidly returned to detectable levels within 10 s of photobleaching in situ, reaching close to its maximum levels by 50 s, with the granular structure forming at the original spot (Figure 6G; Figure S7C and Movie S3,S4, Supporting Information). This relatively fast restoration of mKate‐LSM14B aggregates after photo bleaching suggested that these LSM14B condensates likely formed through liquid‐liquid phase separation. Therefore, LSM14B regulates the fate of maternal mRNAs and oogenesis by forming dynamic and versatile complexes with proteins that participate in mRNA metabolism and oocyte development.

## 3 Discussion

**Figure 7:**
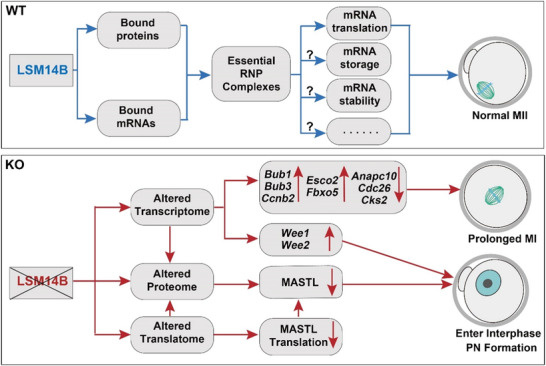
Schematic illustration of the main observations and potential mechanisms of LSM14B in the regulation of maternal mRNA metabolism and oocyte development in the mouse. Question marks in the top panel indicate the other potential mRNA metabolic processes that are regulated by the LSM14B‐directed RNP complexes.

Through identification and characterization of the RBPs expressed in mouse oocytes, this study expanded the known mouse oocyte RBPome, and discovered that LSM14B is an oocyte‐specific RBP that functions in mouse oocytes to regulate the fate of maternal mRNAs and oogenesis. The mRNA‐ and protein‐ interacting partners of LSM14B were identified, and this study showed that LSM14B forms dynamic and versatile complexes with proteins and mRNAs that participate in mRNA metabolism and oocyte development (see **Figure** **7** for a summary).

The oocyte‐exclusive expression and function of LSM14B, versus the more somatic‐restricted expression of LSM14A found here, not only clarified the role of LSM14 in oogenesis but may also help to explain why LSM14 evolves into LSM14A and LSM14B paralogs in vertebrates. Although it was known in oocytes of the lower animal species for >20 years,^[^ ^39^ ^]^ the function of LSM14 in oogenesis was unknown until the current study. In conjunction with the embryonic lethality phenotype observed by others in the *Lsm14a*–KO mice,^[^ ^40^ ^]^ the broad expression of LSM14A shown here suggests that it functions in the somatic cells. It is therefore plausible that as species evolved into the vertebrates, LSM14B became oocyte‐specific and replaced the originally defined oocyte‐specific functions that were operative in its ancestors. At the same time, LSM14A assumed a more diversified expression to support a function in somatic cells. The divergence of such an important LSM14 protein into two paralogs could reflect an evolutionary pressure exerted by the complexity of the development of the distinct somatic and germ cell lineages in the more advanced species to drive the lineage‐specific spatiotemporal regulation of mRNA metabolism.

Essential oogenic regulators have been identified in previous studies and are here referred to as “star factors” of oogenesis. The binding of LSM14B to the proteins and/or mRNAs of most of these “star factors” (e.g., GPR3, MARF1, MOS, NLRP5, NOBOX, NPM2, PADI6, PDE3A, YBX2, and ZAR1) (see Table S2, Supporting Information, for a more extensive listing) suggests that it may serve as a facilitator of these “star factors” to orchestrate mammalian oogenesis. The central role of LSM14B in oogenesis was also supported by its interaction with the mRNAs and/or proteins that are involved in several aspects of the control of oogenesis. The binding of LSM14B to mRNAs for transcriptional factors, epigenetic regulators and factors of RNA metabolism and translation suggests that it may participate in oocyte gene expression at various levels. While the binding to mRNAs for cell cycle regulators, key ODPFs and signaling mediators of oocyte‐granulosa cell communication indicates engagement of LSM14B in regulation of oocyte meiotic progression and follicular development. Moreover, LSM14B bound to both the mRNAs and proteins of most of the components of the oocyte SCMC, a functionally conserved multiprotein complex assembled in the oocyte subcortical regions that is essential for the oocyte‐to‐embryo transition (OET).^[^ ^41^ ^]^ This suggests that LSM14B is involved in regulating the expression and function of the SCMC components. Components of the SCMC are encoded by maternal effect genes, deletions, or mutations of them compromise OET, and cause female‐specific infertility or subfertility and reproductive disorders in mice and humans.^[^ ^41^, ^42^ ^]^ Although SCMCs are known to play diverse roles during OET, the detailed molecular mechanisms underlying the dynamic assembly and function of this maternal proteinaceous structure are not known. The observations put forward here therefore open new avenues for unraveling these mechanisms.

LSM14B interacted with many RBPs involving virtually every process of RNA metabolism, suggesting its engagement in the formation of versatile RNP complexes to regulate maternal mRNA metabolism. Of particular importance is its binding to CPEB, DDX6, EDC4, EIF4E1B, EIF4ENIF1 (4E‐T), MARF1, PABPC1L (ePAB), PATL2, ZAR1, and YBX2, and colocalization with DDX6 in the P‐body‐like structure in non‐growing primordial follicle‐stage oocytes. YBX2 is a well‐known germ cell specific RBP crucial for stabilization and storage of oocyte mRNAs before maturation,^[^ ^10^ ^]^ while the other proteins are involved in translational repression and degradation of mRNA. The association with these RBPs suggests that LSM14B participates in maternal mRNA storage, translational repression and turnover in mouse oocytes, as has been proposed previously for LSM14A in the *Xenopus* oocyte.^[^ ^11^ ^]^ However, a prominent increase in the translation of the LSM14B‐bound mRNAs in the *Lsm14b*‐KO oocytes was not observed in the present study, rather there was a greater number of the LSM14B‐bound mRNAs with the translational efficiency significantly decreased. Moreover, LSM14B bound dozens of ribosomal proteins and translation‐initiating factors. These results together indicate that the predominant role of LSM14B in oocytes is probably not repression of translation, but rather selective promotion of the translation of certain class of mRNAs. A similar assumption was proposed previously for *Xenopus* oocytes.^[^ ^43^ ^]^ The association of LSM14B with the recently identified oocyte‐specific ribonuclease, MARF1, but not with DCP1 and 2, suggests that LSM14B may participate in the regulation of maternal mRNA turnover via an oocyte‐specific mechanism that is independent of decapping.^[^ ^5b–d^ ^]^ The exact role of LSM14B in the regulation of oocyte mRNA metabolism is likely context dependent and awaits further investigation.

The only clear phenotypic effect of the absence of LSM14B was inflicted on oocyte meiotic maturation and early embryogenesis despite its expression throughout oocyte growth and development and association with “star factors.” The slight decrease in the number of ovulated eggs may reflect a perturbation of processes involved in follicular development. More detailed analysis of the dynamics of oocyte and follicular development in the mutant ovaries could demonstrate subtle effects on these processes in future studies.

Cheng et al. recently reported that LSM14B, together with DDX6, EIF4ENIF1, ZAR1, and YBX2, locates in MARDO, a mitochondria‐associated ribonucleoprotein domain existing in FGOs of mouse and several other mammalian species and functioning to store maternal mRNAs.^[^ ^44^ ^]^ MARDO assembles during oocyte growth and becomes most prominent in FGOs, stages when P‐body‐like structure diminishes and disappears.^[^ ^37^ ^]^ Our observation that LSM14B colocalized with DDX6 in P‐body‐like structure in the non‐growing primordial follicle‐stage oocytes thus agrees with its localization in MARDOs during the later stages of oocyte development. These results together provide a more complete view of the spatiotemporal changes in the localization of the LSM14B‐cotaining RNP complexes throughout oocyte development. Whether and how depletion of Lsm14b affects the integrity and function of oocyte MARDO remains to be explored.

## 4 Conclusions

This study shows that the essential oogenic regulators are integrated to control the fate of maternal mRNAs, and to enable meiotic maturation and probably other processes during oogenesis, as indicated by the diverse associations of LSM14B. Given that mutations of genes encoding certain RBPs cause oocyte maturation defects or premature ovarian insufficiency in human,^[^ ^6^, ^18^, ^45^ ^]^ this study may also have implications for the diagnosis and treatment of infertility resulting from the decline of oocyte quality under deleterious conditions such as genetic malfunctions and aging.

## 5 Experimental Section

### Laboratory Mice

Mice carrying a targeted knockout first, reporter‐tagged insertion with conditional potential allele of Lsm14b, Lsm14b^tm1a(KOMP)Mbp^, were obtained from the Wellcome Trust Sanger Institute and were maintained on identical C57BL/6J genetic backgrounds. Lsm14b^tmla/tm1a^ (hereafter referred to as Lsm14b‐KO) mice were produced by mating Lsm14b^tm1a/tm1a^ males with Lsm14b^tm1a/+^ females. Mice were genotyped by PCR using primers as shown in Table S1, Supporting Information, which produces a 652 and 270 bp PCR product for the wild type (WT) and KO alleles, respectively. Female fertility test was carried out by mating with normal adult C57BL/6J males for at least 8 months. A total of 4 WT, and 6 Lsm14b‐KO females were fertility tested. All mice were raised under the standard conditions at the animal facility of Shandong university. All mouse procedures and protocols applied in this study were approved by the Institutional Animal Care and Use Committee of Shandong university.

### Chemicals and Reagents

Unless otherwise specified, all chemicals and reagents were purchased from Sigma‐Aldrich (USA). AZ3146 (Cat No. HY‐14710) and PD166285 (Cat No. 3785) were purchased from MedicalChemExpress (Shanghai, China) and Bio‐Techne/TOCRIS (Minnesota, USA), respectively. Antibodies used in this study were shown in Table S3, Supporting Information.

### Oocyte Isolation and Culture

Oocyte isolation and in vitro maturation were carried out in MEM medium with Earles’ salts (Thermo Fisher Scientific, USA), supplemented with 75 µg mL^−1^ penicillin G, 50 µg mL^−1^ streptomycin sulfate, 0.23 mm pyruvate, and 3 mg mL bovine serum albumin using exactly the same procedure as described previously.^[^ ^46^ ^]^ To study the ability of oocytes to resume and complete the first meiosis, and the kinetics of oocyte meiotic progression, FGOs were matured in MEM medium for various hours. Oocyte culture was carried out at 37 °C and 100% humidity in an Esco CelCulture CCL‐170T‐8‐IVF incubator (Kringelled, Denmark) infused with 5% O_2_, and 5% CO_2_.

### Oocyte In Vitro Fertilization and Embryo Culture

Mature MII‐stage eggs were collected from oviductal ampulla of the mice that were initially primed with eCG for 48 h followed by priming with human chorionic gonadotropin (hCG, Ningbo, China) for 14 h. For In Vitro Fertilization (IVF), the ovulated oocytes were inseminated with normal sperm isolated from B6D2F1 adult males. The formation of pronuclear and 2‐cell stage embryos was recorded 8 and 24 h after IVF, respectively. The 2‐cell embryos were thereafter transferred into KSOM medium for further culture to the blastocyst stage. The formation of 4‐cell, morula, and blastocyst stage embryos was monitored on day 3, day 4, and day 5 after IVF, respectively.

### mRNA Interactome Capture

mRNA interactome capture (RIC) was carried out following the protocol as described previously by Castello et al. (2013).^[^ ^7^ ^]^ This approach of RIC combines UV cross‐linking and oligo(dT) capture to pull down the RBPs bound to mRNA in live oocytes. The captured RBPs are eventually characterized by quantitative mass spectrometry (MS). To do so, GV‐stage oocytes were isolated from eCG primed (46 h) 22‐day‐old mice and collected in MEM‐alpha containing 5 µm Milrinone to prevent resumption of meiosis. The oocytes were washed three times with cold PBS‐PVA buffer, and then immediately irradiated on ice by 254 nm UV light at 15mJ cm^−2^ for 1 min. These UV‐light treated oocytes were instantly collected in 1.5 mL centrifuge tubes, frozen with liquid nitrogen and stored at −80 °C. A total of 50 000 oocytes were collected for two independent experiments, with each replicate using 25 000 oocytes. The oocytes were lysed in lysis buffer (20 mm Tris‐HCl‐pH7.5, 500 mm LiCl, 0.5% LiDS, 1 mm EDTA, 5 mm DTT) for 10 min at 4 °C, and the resultant lysates were incubated by gentle rotation with 2 mL of oligo (dT)_25_ magnetic beads (S1419S, New England Biolabs, USA) for 1 h at 4 °C. The lysates with the beads were then placed on the 50 mL magnetic Separation Rack (New England Biolabs) for capturing for 30 min, with intermittent inversion for several times. After being completely captured, the beads were washed six times with wash buffer, twice with high‐salt wash buffer (20 mm Tris‐HCl‐pH 7.5, 500 mm LiCl, 0.1%LiDS, 1 mm EDTA, 5 mm DTT), twice with buffer containing 20 mm Tris‐HCl (pH 7.5), 500 mm LiCl, 1 mm EDTA, 5 mm DTT, and twice with low‐salt buffer (20 mm Tris‐HCl‐pH 7.5, 200 mm LiCl, 1 mm EDTA, 5 mm DTT). The captured proteins and RNAs were eluted with elution buffer containing 20 mm Tris‐HCl (pH 7.5) and 1 mm EDTA for 3 min at 55 °C. To release proteins, the 500 µL eluate was incubated with 1 µg of RNase A (ST576, Beyotime, China) in 50 µL of 10× RNase buffer containing 100 mm Tris‐HCl (pH 7.5), 1.5 m NaCl, 0.5%NP‐40, and 5 mm DTT for 1 h at 37 °C. Proteins were concentrated using an Amicon Ultra‐0.5 Centrifugal Filter Unit with Ultracel‐3 membrane (UFC500324, Millipore, USA) and sent to the Proteome Core Facility at Nanjing Medical University for LC‐MS/MS. Partial protein sample was saved and used for silver staining.

### LC‐MS/MS Analysis

Liquid chromatography‐tandem MS (LC‐MS/MS) analysis was performed as described previously on EASY nLC 1000 (Thermo Fisher Scientific) and LTQ Orbitrap Velos (Thermo Fisher Scientific) according to the manufacturer's instruction.^[^ ^46^ ^]^ The LC gradient was formulated with MS buffer A (water with 0.1% FA) and buffer B (ACN with 0.1% FA). The analytical column (75 µm × 15 cm) was used in the chip LC with 2 µm 100 Å Acclaim PepMap100 C18 column. Samples were injected and separated with a linear gradient of 3%–100% buffer B over 95 min at a flow rate of 0.3 µL min^−1^. Parameters of the MS survey scan were set with the spray voltage of 1.9 kV, and the quality scan range from 350 to 1800. Peptide analysis was performed in data‐dependent acquisition mode, with the top 20 most intense precursor ions selected for subsequent MS/MS. Orbitrap was used for parent ion scan, with the resolution set at 60 000. Daughter ion scan (i.e., MS2 sequencing) was performed using collision‐induced dissociation (CID) in the ion trap. A normalized collision energy of 35% was used. The mass spectrometric raw data were searched against the mouse UniProt sequence database using MaxQuant version 1.5.2.8 with essentially the default parameters. Enzyme specificity was set to trypsin and up to two missed cleavage sites were allowed. The identified minimal peptide length was set to six amino acids. Cysteine carbamidomethylation (C_2_H_3_NO, +57.0215 Da) was set as fixed modification, and methionine oxidation (+15.9949 Da) and protein N‐terminal acetylation (C_2_H_2_O, + 42.0106) were used as variable modifications. The oocyte RBPome data were deposited to the ProteomeXchange Consortium, with the assigned dataset accession number PXD034071.

### RNA‐Seq Analysis

RNA‐Seq analysis was carried out on three total RNA or polysome‐bound mRNA samples derived from WT and Lsm14b‐KO oocytes as described previously.^[^ ^47^ ^]^ Total RNA was extracted from 20 GV‐stage FGOs, while polysome‐bound mRNA was prepared in the polysome profiling experiment as described in the following section. The differentially expressed transcripts were calculated using default parameter of cuffdiff (v2.2.1), and the significantly changed transcripts were defined by the criteria of FDR *p* < 0.05. RNA‐Seq data were deposited in the Gene Expression Omnibus (datasets GSE204773). Bioinformatics analysis of differentially expressed transcripts was conducted using Metascape (http://metascape.org).

### Proteomic Analysis

Microproteomic technology for single cell or low‐input samples was used to study the proteomic changes in Lsm14b‐KO oocytes. Triplicate samples of WT and Lsm14b‐KO oocytes, with each having 20 GV‐stage FGOs, were collected with 1 µL PBS into 0.2 mL low protein binding PCR tubes that were pre‐coated with 0.2 µg µL^−1^ synthetic peptide (FFWIKVFFIK VFFVKIFFVKIFFVKIFFVK). These microsamples were suspended with appropriate amount of 50 mm ammonium bicarbonate buffer containing 5 mm DTT and sonicated in a water bath at 37 °C for 15 min. The cell lysate was then alkylated with 10 mm IAM in dark for 30 min, digested with 1 µg µL^−1^ trypsin at 37 °C for 2 h, and finally subjected to LC–MS/MS. Simultaneously, proteins from 1000 GV‐stage oocytes (library sample) were extracted with lysis buffer (7 m urea, 2 m thiourea, 0.2% SDS, 20 mm Tris, pH 8.0–8.5) supplemented with 1 mm PMSF, 2 mm EDTA and complete protease inhibitor cocktail for constructing the spectral library.

LC–MS/MS were performed on a Fusion Lumos Tribrid mass spectrometer (Thermo Scientific) equipped with an UltiMate 3000 UHPLC (Thermo Fisher Scientific). Peptides were loaded on the analytic column (length, 30 cm; inner diameter, 150 µm) packed in house with 1.7 µm C18 particles, and eluted at a flow rate of 500 nL min^−1^ with a gradient of 5%–25% buffer B (0.1% formic acid in 98% acetonitrile) over 90 min, 25%–35% buffer B over 10 min, and 35%–80% buffer B over a subsequent 5 min. MS data was acquired in DDA mode, with the scan parameters set at a resolution of 2 40 000 (MS1) and 30 000 (MS2), an automatic gain control (AGC) target of 4e5 (MS1) and 5e4 (MS2), a maximum injection time (IT) of 100 ms (MS1 and (MS2), and a scan range of 350–1500 m z^−1^ (MS1) and 1.6 m z^−1^ (MS2), and a first mass of 100 for MS2. For the 1000‐oocyte library sample, the MS1 scan parameters were set at resolution of 1 20 000, AGC target of 1e5, maximum IT of 50 ms, and scan range of 350–1500 m z^−1^. The MS2 scan parameters were set as follows: resolution, 30 000; AGC target, 2e4; maximum IT, 50 ms; Top 20; and isolation window, 1.6 m z^−1^. Unless otherwise specified, all the other parameters were set as default.

Raw data were processed by MaxQuant (version1.5.3.30) for feature detection, database searching, and protein quantification with the Swiss‐Prot mouse database. Oxidation (M), acetylation (protein N‐term), carbamidomethylation (C), deamidation (NQ) and Gln→pyro‐Glu were set as variable modifications. Protein identification was achieved by using the MaxQuant's integrated Andromeda engine. The identification results of the micro‐sample data were filtered at the spectrum level with PSM‐level FDR < = 1%, and then further filtered at the protein level with Protein‐ Level FDR < = 1% to obtain significant identification results. For protein quantification and difference analysis, MaxQuant was used to extract peak areas and calculate protein quantitation values. Then, according to the set comparison groups, the multiples of differences in the proteins in each comparison group were calculated, and the significance test was performed using Welch's *t*‐test. Furthermore, screening was performed based on the multiple of difference >1.2 and *p*‐value < 0.05 as the criteria for determining significant differences. Proteomic data have been deposited in the Proteome Xchange Consortium, with the assigned dataset accession number PXD034070.

### LACE‐Seq

GV‐stage FGOs were isolated from the ovaries of eCG primed (46 h) 22‐to 23‐day‐old mice for LACE‐Seq. Oocytes were washed three times with cold PBS‐PVA buffer, and immediately irradiated on ice with UV light at 400 mJ cm^−2^ for two times. The UV light treated oocytes were stored at −80 °C until 2000 oocytes required for one experiment were accumulated. LACE‐Seq was carried out as described previously,^[^ ^17^ ^]^ with the same experiment repeated independently twice. LACE‐seq data were deposited in the Gene Expression Omnibus, with the assigned dataset accession number GSE206190.

### Polysome Profile Analysis

Polysome profile analysis was carried out as described previously^[^ ^48^ ^]^ with slight modifications. Briefly, 1500 WT and Lsm14b KO oocytes were lysed, respectively, in polysome lysis buffer (100 mm KCl, 0.1%Triton X‐100, 50 mm HEPES, 2 mm MgCl2, 10% glycerol, 100 µg mL^−1^ cycloheximide, 1 mm DTT, 20 unit mL^−1^ EDTA‐free RNase inhibitor, and EDTA‐free proteinase inhibitor cocktail). Lysates were loaded onto 15%–55% (w/v) sucrose density gradients prepared with 10 mm Tris‐HCl (pH 7.5), 5 mm MgCl2, 100 mm NaCl, and 1 mm DTT, and centrifuged at 38 000 rpm for 3 h at 4 °C in a Beckman SW41Ti rotor. Gradients were fractionated and the absorbance at 254 nm was continuously recorded using Gradient Fractionator (BioComp, Canada). The mRNA in the polysome fraction (i.e., 11–28^th^ fractions) were extracted using the RNeasy Micro kit (Qiagen, Germany) and sent for RNA‐Seq analysis (See the later section). The Polysome‐Seq data were deposited in the Gene Expression Omnibus, with the assigned dataset accession number GSE206270.

### Co‐Immunoprecipitation

A total of ≈8000 GV‐stage FGOs were lysed using the lysis buffer from the Pierce Crosslink Immunoprecipitation Kit (Cat No. 26147, Thermo Fisher Scientific). Immunoprecipitation (IP) was then carried out on these oocyte lysates using the anti‐LSM14B antibody. The IP products were subjected to WB validation followed by Mass Spectrometry (MS) analysis. For MS analysis, the IP products from two independent experiments were resolved simultaneously on 10% SDS‐PAGE, and the lanes corresponding to each IP product were sliced out and sent to the Proteomics Core Facility of Nanjing Medical University (Nanjing, China) for subsequent analysis. The gel slices were cut into 1 mm^3^ particles at the Core Facility, destained, reduced, and alkylated, followed by the overnight Trypsin in‐gel digestion at 37 °C. LC‐ESI‐MS/MS analysis was performed using a Ekspert nano LC 415 system and a TripleTOF 5600 mass spectrometer (AB Sciex), respectively. The raw data were analyzed using MaxQuant (version 1.5.2.8), with the UniProt Mouse database as reference. Data were searched using the following parameters: trypsin as the enzyme; up to two missed cleavage sites were allowed; 10 ppm mass tolerance for MS and 0.05 Da for MS/MS fragment ions; propionamidation on cysteine as fixed modification; oxidation on methionine as variable modification. The incorporated Target Decoy PSM Validator in Proteome Discoverer and the Mascot expectation value was used to validate the search results and only the hits with FDR ≤0.01 and MASCOT expected value ≤0.05 were accepted for discussion. Co‐IP data were deposited in the ProteomeXchange Consortium with the assigned dataset accession number PXD034185.

### qRT‐PCR Analyses

For qRT‐PCR analysis, total RNA was isolated from oocyte samples using the Fast Pure Cell/Tissue Total RNA Isolation Kit (RC101‐01, Vazyme, China), and reversed transcribed by the HiscriptIII RT Supermix for qPCR (R323‐01, Vazyme). AceQ Universal SYBR Master Mix (Q511‐02, Vazyme) was then carried out using primer pairs shown in Table S1, Supporting Information. Relative changes in mRNA levels between WT and KO oocytes were analyzed by the 2^−ΔΔCt^ method using Rpl19 as internal control, while exogenous mKate mRNA was used as the normalizing control for comparison between the same number of FGOs and preimplantation embryos.

### Preparation of mRNAs for Microinjection

A live‐imaging and fluorescence reporter‐based 3′UTR assay strategy was adopted to assess the effect of LSM14B binding to the 3′UTR of Mastl mRNA on its translation.^[^ ^49^ ^]^ To prepare the construct for this assay, the sequence of fluorescent protein mKate was first amplified by PCR using the pCMV6‐AN‐mKate plasmid DNA as template, and cloned into pCMV6‐AC‐3DDK (Origene, USA) vector. The resulted pCMV6‐AC‐mKate‐3DDK construct was then used as the destination vector for cloning the Mastl‐3′UTR. Mastl‐3′UTR was amplified by PCR using the mouse oocyte cDNA as template, and inserted into the pCMV6‐AC‐mKate‐3DDK vector at the site immediately downstream of the stop codon of 3DDK. Deletion of the LSM14B binding site (TAGTGATGGAGTCTCACTGC) within Mastl‐3′UTR was achieved by using the Mut Express MultiS Fast Mutagenesis Kit V2 (Vazyme, China) as tool and the pCMV6‐AC‐mKate‐3DDK‐Mastl‐3′UTR plasmid DNA as template. EGFP sequences were also cloned into the pCMV6‐AC‐3DDK at the site upstream of 3DDK and served as the template for synthesizing control EGFP mRNA. Then, sequences of the complete or LSM14B‐binding site‐deleted Mastl‐3′UTRs were amplified by PCR using a pair of primers that flank the T7 promoter and the 3′‐end of Mastl‐3′UTR. The reverse primer had a stretch of 20 Ts added at its 5′‐ end in order to make the synthesized mRNA having a short oligo A tail. In vitro transcription was finally carried out on these PCR products using the Ambion's mMESSAGE mMACHINE kit (Thermo Fisher Scientific), and the resulted mRNAs were purified by lithium chloride.

To prepare the construct for synthesizing Mastl mRNA, Mastl CDS was amplified by PCR using the mouse oocyte cDNA as template, and inserted into the pCMV6‐AC ‐3DDK vector at the site immediately upstream of the 3DDK. Plasmid DNA of this resulted pCMV6‐AC‐Mastl‐3DDK construct, as well as those for GFP‐tagged tubulin, mCherry‐tagged H2B, Venus‐tagged CyclinB1, and mCherry‐tagged Securin, were linearized and transcribed in vitro using the same Ambion's mMESSAGE mMACHINE kit as mentioned above. The transcribed mRNAs were then polyadenylated in vitro using the Poly(A) Tailing Kit (Thermo Fisher Scientific), and purified by lithium chloride.

### Microinjection of Oocytes with mRNA

The mRNA of purified EGFP and the complete or LSM14B‐binding site‐deleted Mastl‐3′UTRs were mixed to make a final concentration of 100 and 500 ng µL^−1^, respectively. The mixed mRNAs were then micro‐injected into WT‐GV‐stage FGOs in M2 medium containing 5 µm milrinone. ≈10 pL of the mRNAs were injected into the cytoplasm of 1 FGO. The injected oocytes were first incubated for 12 h in milrinone medium to allow the mRNAs to express to levels optimal for fluorescence detection, then the expression of mKate and EGFP was recorded under a Nikon Ti2‐E‐C2+ confocal microscope (Nikon, Japan).

For live‐imaging experiments, purified mRNAs for the mCherry‐tagged H2B and GFP‐tagged tubulin pair, and Venus‐tagged CyclinB1 and mCherry‐tagged Securin pair were mixed at 1:1 ratio to make a final concentration of ≈500 and 20 ng µL^−1^, respectively for each mRNA, and micro‐injected into the GV‐stage FGOs. Live‐imaging was then carried out as described previously on an Andor spinning‐disk confocal microscope (Andor Technology Ltd, Belfast, Northern Ireland).^[^ ^46^ ^]^ Quantification of the fluorescence intensity of the recorded images was done using the Image J software (http://imagej.nih.gov/ij).

### FRAP Analysis

HEK293T cells were cultured in DMEM (Meilunbio, China) containing 10% fetal bovine serum (FBS) at 37 °C with 5% CO2 in a humidified incubator. Plasmid DNAs of pCMV6‐AN‐mKate‐Lsm14b and pCMV6‐AC‐Ddx6‐EGFP‐3DDK were transfected into these cells using the JetPRIME (Polyplus, France) transfection reagent according to the manufacturer's protocol. Transfected HEK293T cells were cultured for 24 h to let the fluorescence tagged protein express to sufficient levels for further detection. Potential colocalization of these two tagged proteins was then examined under the confocal microscope. To express mKate‐tagged LSM14B (LSM14B‐mKate) protein in the early‐stage growing oocytes isolated from 6 day old mice, the mRNA for LSM14B‐mKate was microinjected into the oocyte cytoplasm, followed by culture of the injected oocytes in MEM medium supplemented with 5% serum for 12 h to allow the LSM14B‐mKate protein expression. For fluorescence protein recovery after photobleaching (FRAP) analysis, rectangular regions of interest (ROI) containing the P‐body‐like granular fluorescence puncta of LSM14B‐mKate were marked and photobleached using the excitation laser line at greater than 50% of the maximum power. Two and 10 photos were taken for the HEK293 cells and oocytes, respectively, before bleaching, and thereafter, images were captured every 2 s. Fluorescence intensities of ROIs over time were quantitated and normalized by subtracting the remaining signal after photobleaching and then normalized to the mean intensity before photobleaching.

### Immunostaining and Confocal Microscopy

These were performed using the same procedures as described previously.^[^ ^47^ ^]^ Briefly, oocytes were fixed in 4% PFA in PBS for 30 min at room temperature, and then permeabilized and blocked for 1 h in PBS containing 0.1% Triton X‐100 and 10% FBS. They were subsequently incubated with primary antibodies (4 °C, overnight) and Alexa flour 594/488‐conjugated secondary antibodies (room temperature, 1 h), respectively. DNA was counterstained with Hoechst 33342 for 30 min. For assessment of the kinetochore–microtubule attachment in oocytes, oocytes that were matured in vitro for 8.5 h were first kept on ice for 10 min (cold treatment), and then subjected to IF staining of the kinetochore and microtubule using the human anti‐centromere and mouse anti‐*α*‐tubulin antibodies, respectively. IF stained oocyte samples were finally mounted in a small drop of antifade on glass slides, and subjected to confocal imaging. For IF staining of the ovarian sections, freshly isolated ovaries from 3‐ or 21‐day‐old female mice were fixed in 4% PFA at 4 °C overnight, and 5 µm thick paraffin sections were then prepared. Sections were incubated with the primary antibodies, followed by Alexa flour 594‐ or 488‐conjugated secondary antibodies, and the DNA was counterstained with Hoechst 33342 for 10 min. Both the oocyte and ovarian specimens were examined and imaged using a laser scanning confocal microscope (Nikon Ti2‐E‐C2+, Nikon).

### Western Blot Analysis

Western Blot analysis was carried out as described previously.^[^ ^46^ ^]^ Briefly, oocyte samples were lysed in 2× Laemmli sample buffer, and boiled at 100 °C for 5 min to be denatured. The protein lysates were resolved on SDS‐PAGE gels and transferred onto polyvinylidene difluoride (PVDF) membranes for probing the proteins under examination. Beta‐actin (ACTB) or GAPDH was selected to serve as internal control of samples.

### Pathway Enrichment Analysis

The differentially expressed genes (proteins or mRNAs) identified by proteomics or RNA‐Seq analyses were uploaded to Metascape (http://metascape.org), a gene annotation & analysis resource, and the significantly associated terms (GO/KEGG terms or canonical pathways) were then calculated automatically.

### Statistical Analysis

Statistical analyses were performed using Graphpad Prism software (Graphpad software, Inc, USA). Data were presented as mean ± SEM of at least three independent experiments. For experiments with only two treatments, differences between them were analyzed by Student's *t*‐test, while for experiments involving more than two treatments, differences between groups were compared by one‐way ANOVA followed by Tukey's Honestly Significant Difference (HSD). *p* < 0.05 was defined as significantly different.

## Conflict of Interest

The authors declare no conflict of interest.

## Author Contributions

H.L. and H.Z. contributed equally to this work. Y.‐Q.S., H.L., and Y.X. conceived the study; H.L., H.Z., R.S., C.Y., M.L., J.L., and L.S. performed the research; H.L., H.Z., Y.X., and Y.‐Q.S. analyzed the data; and Y.‐Q.S., H.L., and Y.X. wrote the paper.

## Supporting information

Supporting Information

Supplemental Movie 1

Supplemental Movie 2

Supplemental Movie 3

Supplemental Movie 4

Supplemental Table 1

Supplemental Table 2

Supplemental Table 3

Supplemental Table 4

Supplemental Table 5

Supplemental Table 6

Supplemental Table 7

## Data Availability

The data that support the findings of this study are available in the supplementary material of this article.
