# Supplementary material for: LSM14B is an Oocyte‐Specific RNA‐Binding Protein Indispensable for Maternal mRNA Metabolism and Oocyte Development in Mice
Source: Adv Sci (Weinh). 2023 Apr 21;10(18):2300043. doi: 10.1002/advs.202300043 (PMC10288277; doi:10.1002/advs.202300043)
Supplement: Supplementary file 1 — Supporting Information [file ADVS-10-2300043-s006.pdf]

ADVANCED SCIENCE
Open Access

Supporting Information

for *Adv. Sci.*, DOI 10.1002/advs.202300043

LSM14B is an Oocyte-Specific RNA-Binding Protein Indispensable for Maternal mRNA Metabolism and Oocyte Development in Mice

Hui Li, Hailian Zhao, Chunhui Yang, Ruibao Su, Min Long, Jinliang Liu, Lanying Shi, Yuanchao Xue and You-Qiang Su**

Supporting Information

LSM14B is an Oocyte-Specific RNA-Binding Protein Indispensable for Maternal mRNA Metabolism and Oocyte Development in Mice

Hui Li#, Hailian Zhao#, Chunhui Yang, Ruibao Su, Min Long, Jinliang Liu, Lanying Shi, Yuanchao Xue, and You-Qiang Su**

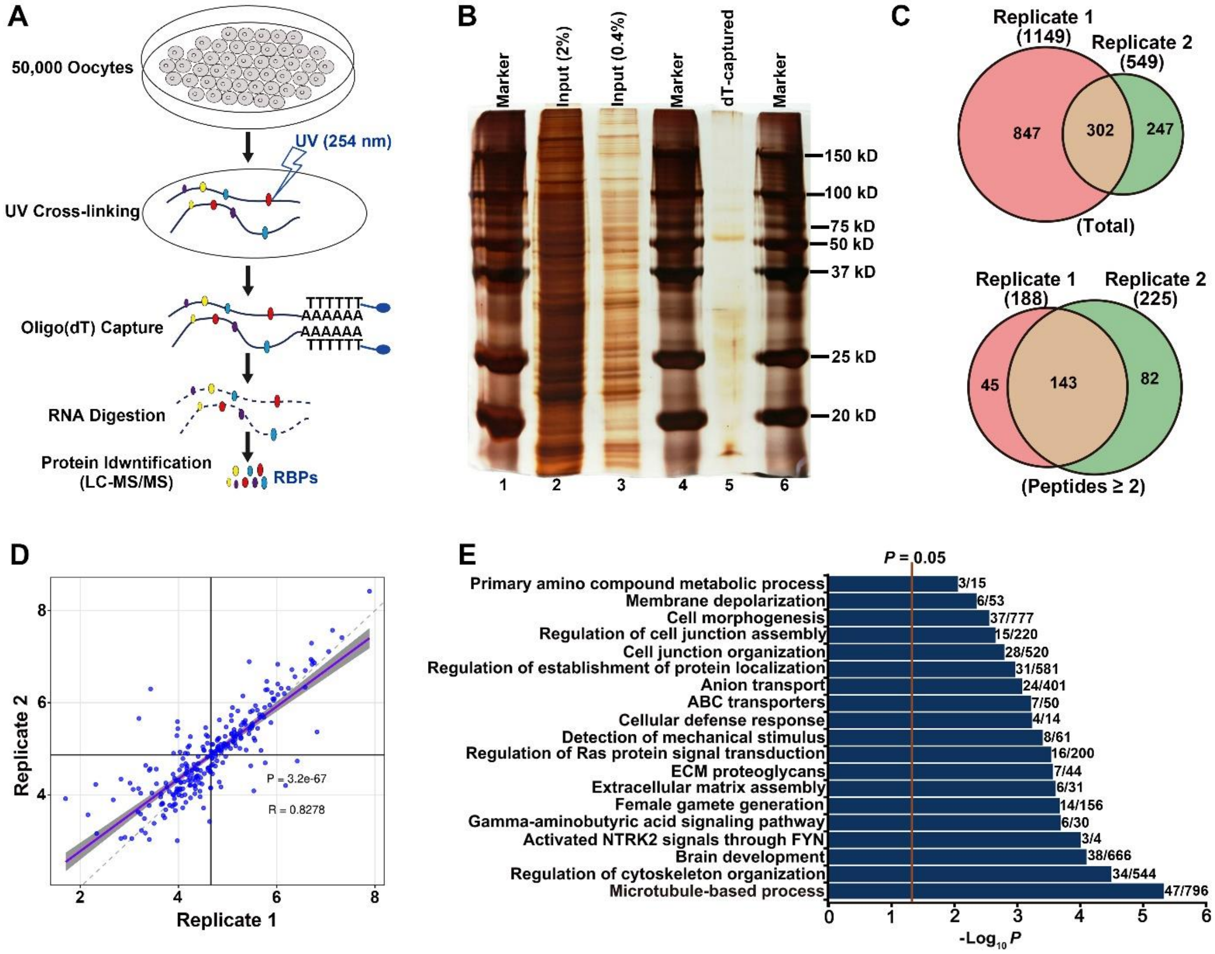

Figure S1. Identification and characterization of the RBPs expressed in mouse oocytes. (A) Schematic illustration of the procedure for mRNA interactome capture (RIC) in oocytes. (B) Silver staining of protein samples before (input) and after RIC (dT-captured) (n=2). (C) Ven diagram illustrating the relationship of the RBPs identifed in the two independent biological replicates. (D) Correlation plot for the intensity (iBAQ) of the RBPs identified in both biological replicates. (E) GO/KEGG terms or canonical pathways associated with the oocyte unknown RBPs that are not present in the RBPbase.

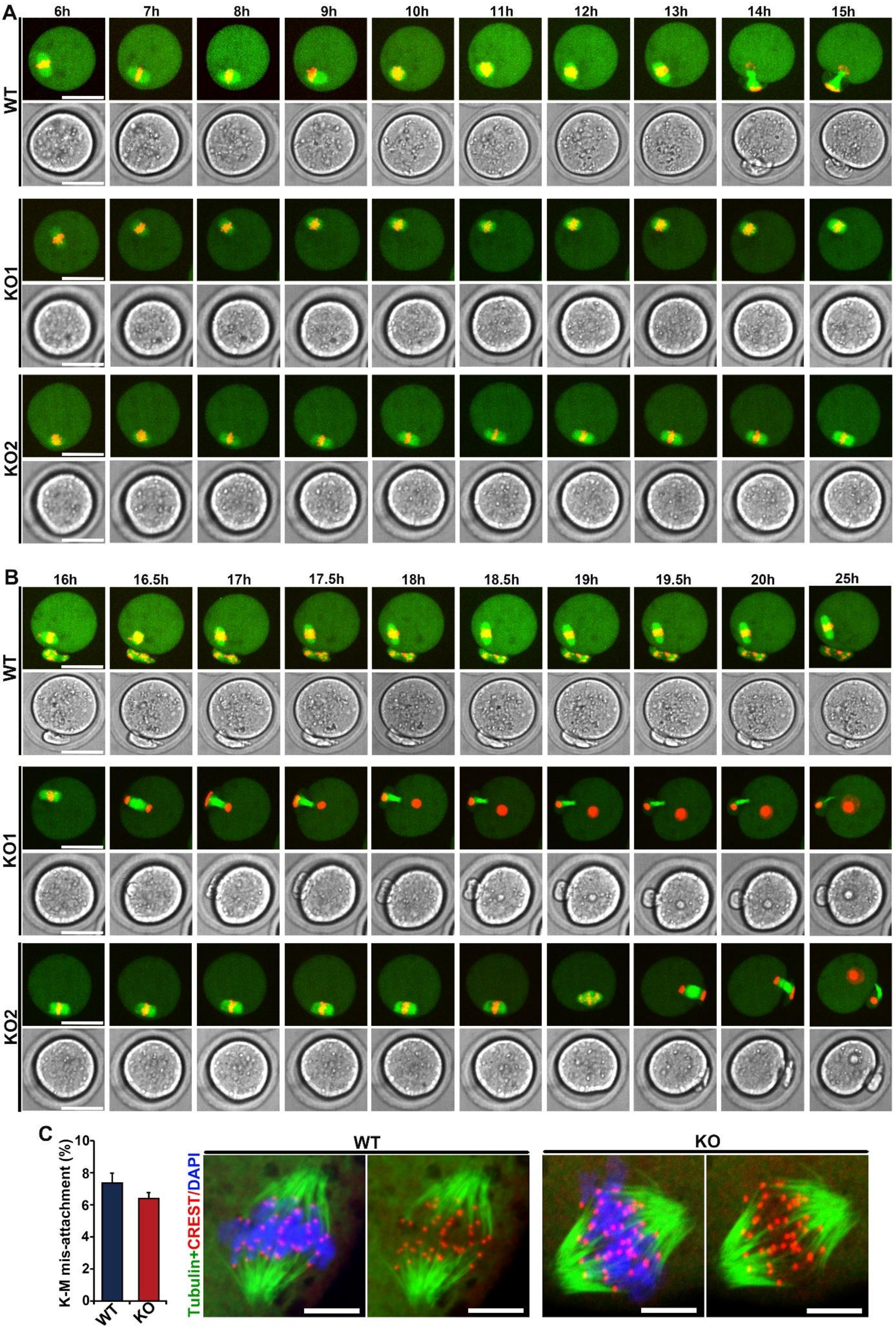

Figure S2. Defects of the *Lsm14b*-KO oocytes in the progression of meiosis to anaphase I and metaphase II. (A, B) Representative still images from the spinning disk confocal live

imaging of the progression of meiosis by WT and *Lsm14b*-KO oocytes in culture (n=2). Time indicates hours after oocytes being released from meiotic arrest and undergoing *in vitro* maturation (IVM). Chromosomes were labeled with H2B-mCherry (shown in red), and meiotic spindles were labeled with β5-tubulin-EGFP (shown in green). Scale bars indicate 50μm. (C) Analysis of kinetochore–microtubule (K-M) attachment in oocytes matured *in vitro* for 8 h by IF staining following cold treatment of the oocyte. Quantification of the percentage of oocytes with mis-attached K-M is shown in the left panel. Data are the mean ± s.e.m (n=3, a total of 25 WT- and 30 KO-oocyte spreads were evaluated). No significant difference between the WTs and KOs by Student's *t*-test. Representative micrographs of the IF staining are shown in the right panel. Microtubules, kinetochores, and chromosomes are stained in green, red, and blue, respectively. Scale bars represent 5μm.

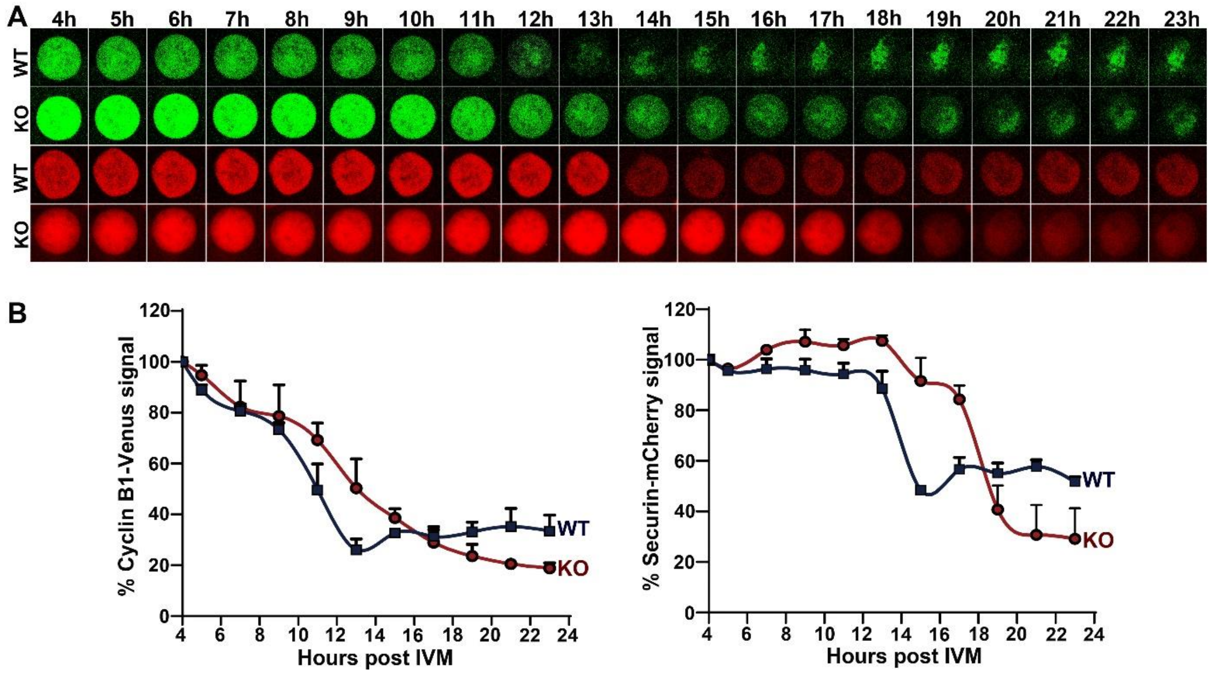

Figure S3. Abnormal activation of anaphase promoting complex (APC) in *Lsm14b*-KO oocytes during metaphase I-to-metaphase II transition. Oocytes were injected with the mRNAs encoding Venus-tagged CCNB1 (Cyclin B1) and mcherry-tagged PTTG1 (Securin) and, after 4h incubation, released into milrinone-free medium. Dynamic changes of the two fluorescent proteins during IVM were monitored via live-cell imaging under a spinning disk confocal microscope. (A) Representative still images of the oocyte after various hours of IVM. (B) Quantification of the dynamic changes of the CCNB1-Venus and PTTG1- mcherry in the oocyte during IVM. Data are the mean ± s.e.m (n=3).

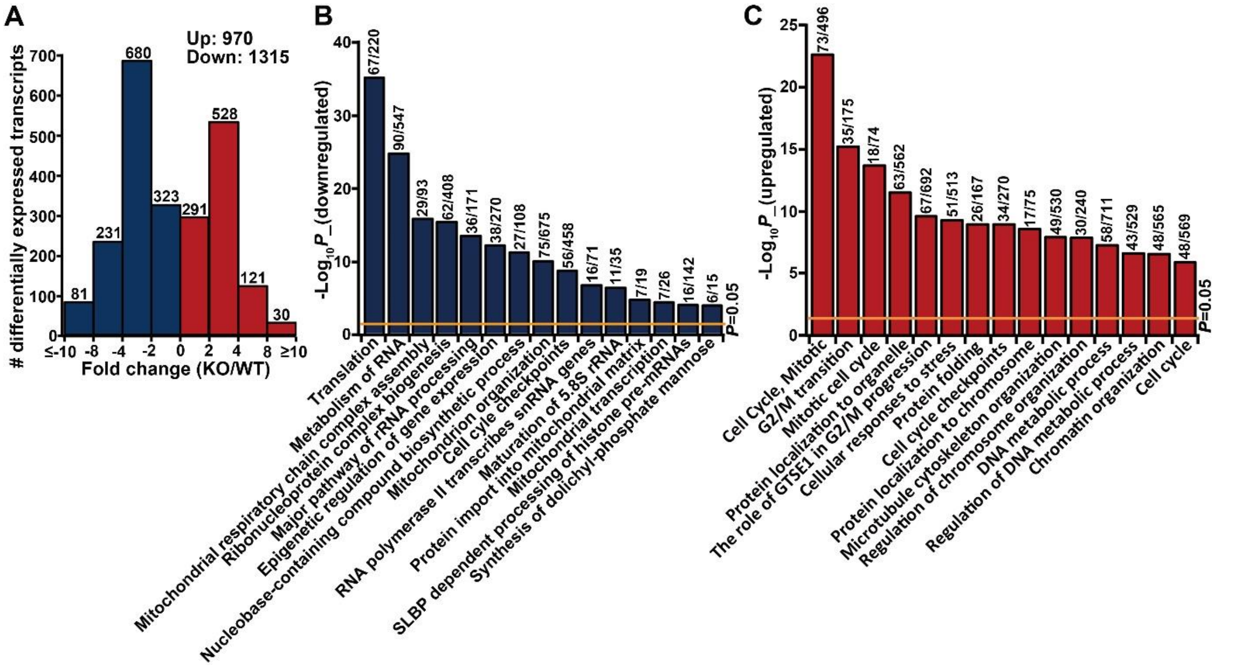

Figure S4. Changes in the transcriptome of the *Lsm14b* KO oocyte. (A) Distribution of the significantly changed transcripts at various levels of fold change. The number of changed transcripts in each category of fold change is indicated above the bars (n=3). (B, C) Bar graphs illustrating the enriched GO/KEGG terms or canonical pathways associated with the significantly downregulated (B) and upregulated (C) transcripts in *Lsm14b* KO GV-stage oocytes, respectively.

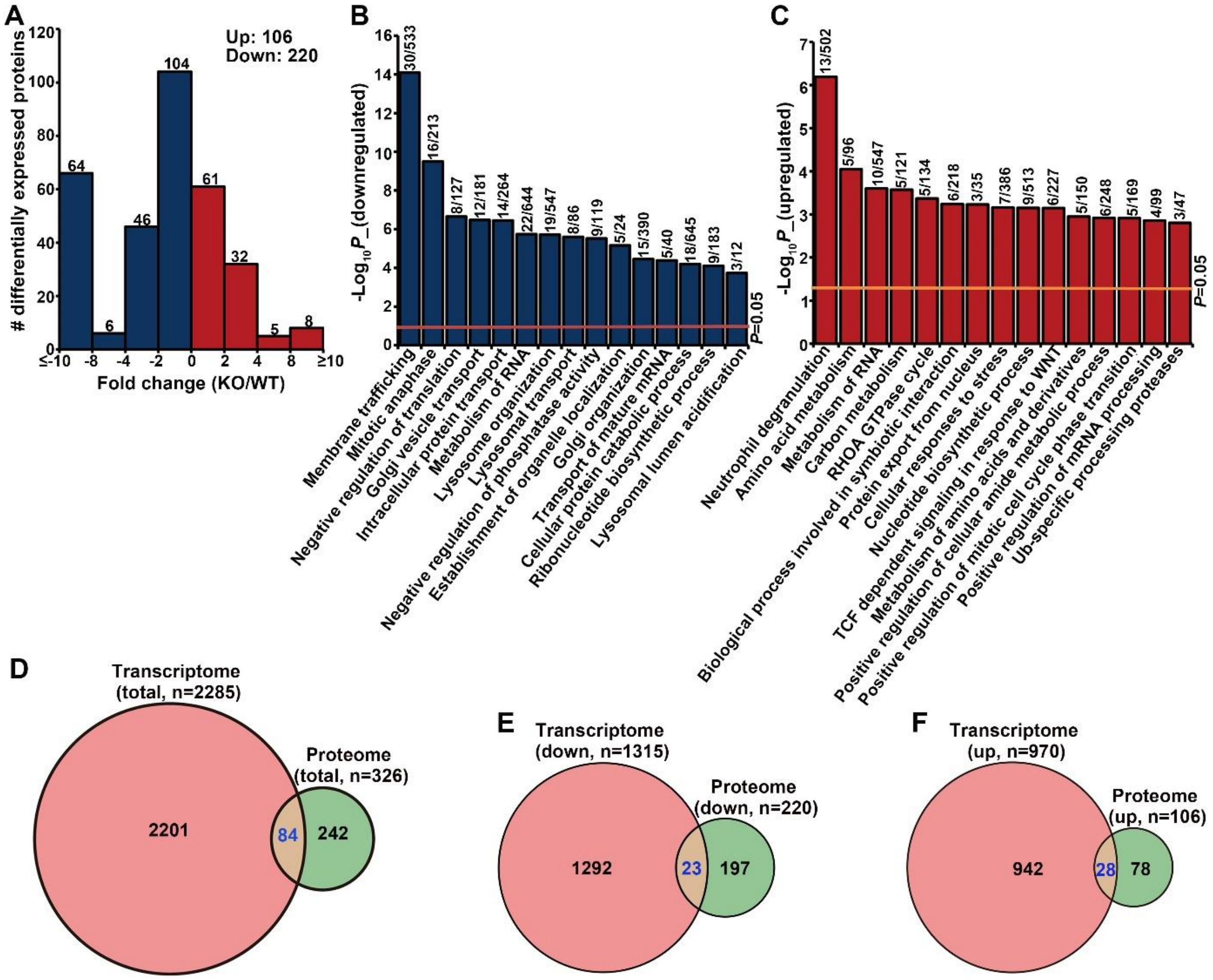

Figure S5. Proteomic analysis of *Lsm14b*-KO oocytes. (A) Distribution of the significantly changed proteins at various magnitudes of difference in expression levels between *Lsm14b*-KO and WT FGOs detected by LC-MS. The number of changed proteins in each category of fold change is indicated above the bars (n=3). (B, C) Bar graphs illustrating the enriched GO/KEGG terms or canonical pathways associated with the significantly down- (B) and up- (C) regulated proteins in *Lsm14b*-KO oocytes, respectively. (D-F) Venn diagrams illustrating the overlap of the transcriptomic changes with those of the proteome in *Lsm14b*-KO oocytes.

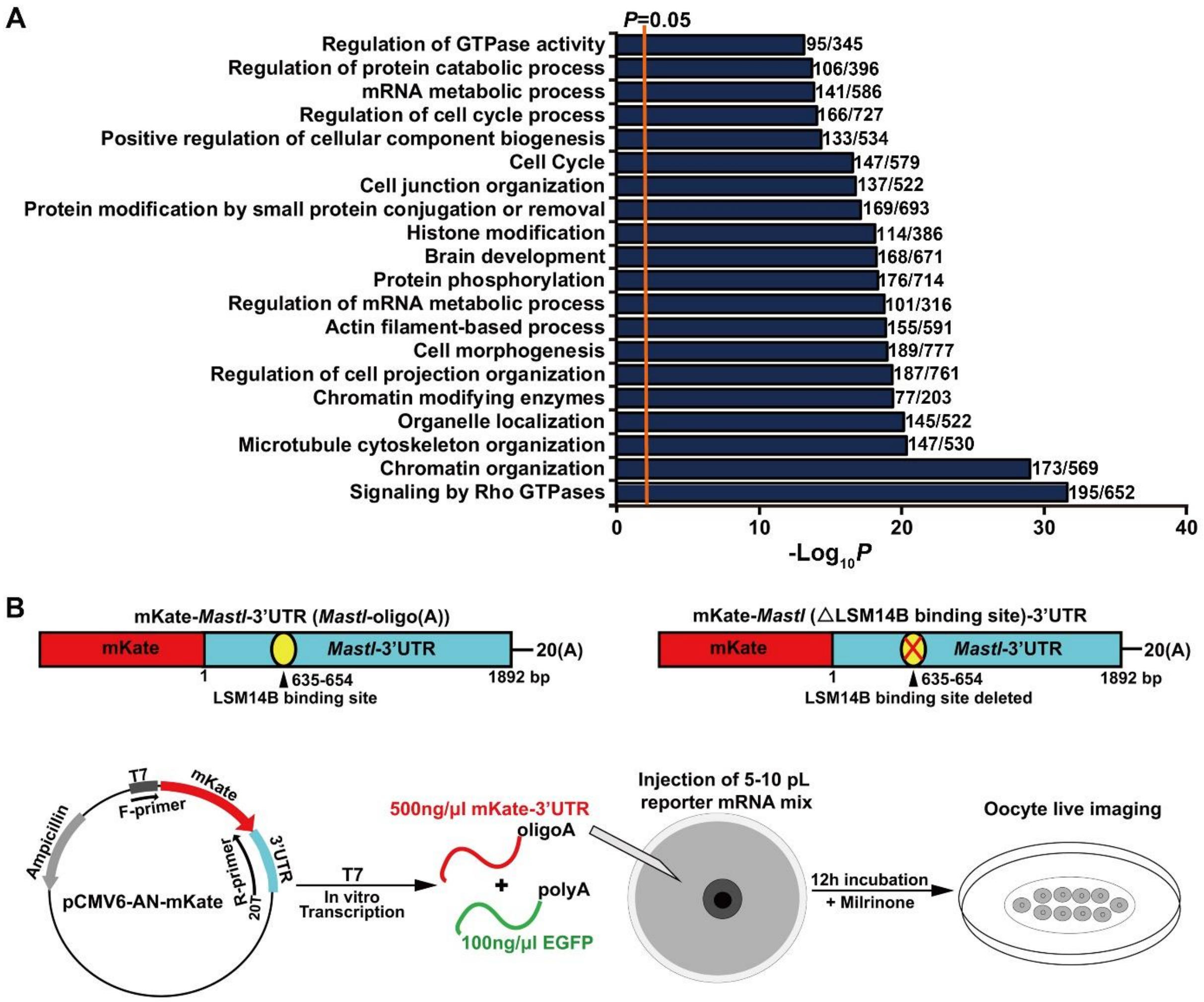

Figure S6. Characterization and validation of the LSM14B-bound mRNAs identified by CLIP and LACE-seq in oocytes. (A) Bar graph illustrating the enriched GO/KEGG terms or canonical pathways associated with the LSM14B-bound mRNAs identified by CLIP and LACE-seq in oocytes. (B) Schematic illustrating the live-cell imaging based 3'UTR-fluorescence reporter assay for evaluating the effect of LSMA4B binding to *Mastl*-3'UTR on *Mastl* translation. The intact or the LSM14B binding site (nt 635-654)-deleted 3'UTR of *Mastl* was cloned into the pCMV6-AN-mKate vector immediate downstream of the mKate sequence. The templates containing the T7 promoter at 5' end and 20 Ts at the 3' end for *in vitro* transcription of mKate-*Mastl*-3'UTR were then obtained by PCR amplification. The mRNAs of mKate-*Mastl*-3'UTR and mKate-*Mastl* (ΔLSM14B binding site)-3'UTR with 20 As at the 3' end were then synthesized *in vitro* using the T7 polymerase. These mRNAs were then mixed with EGFP mRNA that contains a poly(A) tail after purification, and microinjected into the GV-stage oocytes. Live imaging was carried out after the initial incubation of the injected oocytes in milrinone medium for 12h to allow the injected mRNA expressing to a substantial level.

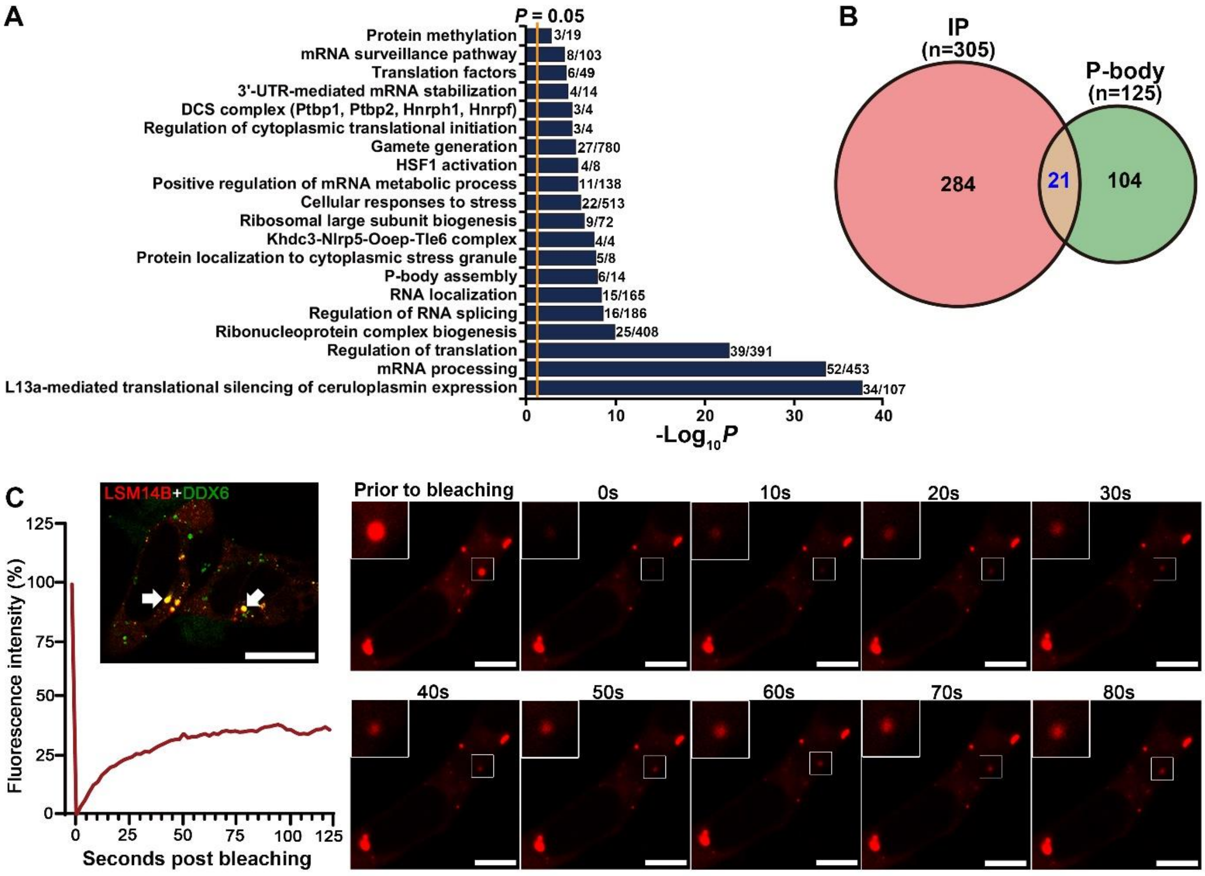

Figure S7. Characterization of the putative LSM14B-interacting proteins identified by CoIP-MS in mouse oocytes, and the phase-separation property of LSM14B formed aggregates in HEK293 cells. (A) Bar graph illustrating the enriched GO/KEGG terms or canonical pathways associated with putative LSM14B-interacting proteins identified by CoIP-MS. (B) Venn diagram illustrating the overlap of LSM14B-bound proteins with the P-body protein components identified by Hubstenberger et al[1]. (C) Live-cell imaging of the expression of mKate-LSM14B (in red) and EGFP-DDX6 (in green) in HEK293 cells after co-transfection with the plasmid DNAs carrying mKate-*Lsm14b* and EGFP-*Ddx6*, and FRAP analysis of the phase-separation property of the LSM14B-mKate-formed P-body-like large fluorescent puncta in live HEK293 cells. Dynamic changes of the fluorescence intensity and morphology of the mKate-LSM14B puncta (in red) before and after photo bleaching are shown in the left bar graph and the right micrographs, respectively. Insets in the up left corner are the magnified views of the boxed area before and after photobleaching. Arrows indicate the LSM14B and DDX6 co-formed P-body-like large puncta. Scale bars represent 5 µm.

Table S1. Primers used for PCR analyses

Gene	Forward primer sequence(5'-3')	Reverse primer sequence(5'-3')	Type of application
Anapc10	TCCAATTCAGAAGAAAAACAACAGT	CCGGATTTCTTGAAGGTTGTGA	qRT-PCR
Bub1	AGGCTCAGCAAGTTATGTACTGC	CTGTCTTCACTAACCCACTGC	qRT-PCR
Bub3	ATTCCATGAGGCTCAAGTACCA	GCATCGTGAGTTCCAACAAGA	qRT-PCR
Ccnb2	CAGTGACTACGTGAAGGACATC	TGGCACGCATACGTCCATTTA	qRT-PCR
Cdc26	CGGAAACCGACCCGCTTAG	ATCGCTGGTTCCTACACCTTC	qRT-PCR
Cks2	GTGATTGGTTGGTGGCTTCG	GACGCAACGACTCGAATGAG	qRT-PCR
Esco2	TCCCTCAGGTGCAGTAGTTTC	TCTTCAGGTCTTTTTGTGTTCACTG	qRT-PCR
Fbxo5	TATTGCCAGCGGGAACTTTAG	GCCAGGATGTCTAGGTGCTC	qRT-PCR
Lsm14a	AGATCCGCTACGAGGGCAT	TCGAGGCGGTATTGGACGA	qRT-PCR
Lsm14b	TGCTATTGTTCAGTCCTCCCTG	CTAGACCTAAGGAAGCGGCAT	qRT-PCR
***Lsm14b*-KO**	TCCAGTTCCATAGCCCTGAAG	TCGTGGTATCGTTATGCGCC	KO allele genotyping
***Lsm14b*-WT**	TCCAGTTCCATAGCCCTGAAG	AGTTCTCCTCTGGCTGGCAC	WT allele genotyping
Mapk3	ACCACATTCTAGGTATCTTGGGT	AGTTTCGGGCCTTCATGTTAAT	qRT-PCR
Mastl	TAGAGGAGTGCGTGAGTAGGA	ACGGCTAATGGGCTTCACTAT	qRT-PCR
Plk1	CCGCAATCAGGTCATTCACA	GCCAAGCCAAAATCCCCTAT	qRT-PCR
Prc1	AACTCACCTCCGGGAAATATGG	GGATATGCTTTTGAGCAGCCT	qRT-PCR
Rpl19	TCAGGCTACAGAAGAGGCTTGC	ATCAGCCCATCCTTGATCAGC	qRT-PCR
Tpx2	CACACCGTTGAAGGCAGTTG	ACAGCTCTCTTAGCATCCAGG	qRT-PCR
Wee1	ACTCCCAAGAGTTTGCTTTCCA	AGGATCCGGAGTAAAGGGGT	qRT-PCR
Wee2	TCTACAAGTGCATTAAGAGGCTG	TAGCGTACCACATGGGGATGA	qRT-PCR

Table S2. Complete list of the "Star factor" of oogenesis identified by LACE-Seq and LSM14B-CoIP-MS and their reported functions in mouse oocytes

Categories of the "Star Factors"	Name of the "Star Factors"	Phenotypes in KO mice or functions in mouse oocyte[references]
RBPs involving RNA metabolism and translation	BTG4	Infertility; Early embryonic developmental arrest[2]
	CNOT6L	Infertility; Meiotic cell cycle arrest at prometaphase I[3]
	CPEB1	ZP3-promoter driven transgenic RNAi causes oocyte and follicle development defects and infertility[4]; KO: Arrest of oogenesis at embryonic day 16.5 (E16.5), when most oocytes are in pachytene of prophase I[5]
	DAZL	Defective spindle formation (Morpholino-KD)[6]
	DDX6	Impaired primordial follicle formation and oocyte growth[7]
	ELAVL2	Infertility; Defective primordial follicle formation[7]
	ESRP1	Infertility; Meiotic defects[8]
	IGF2BP2	Infertility; 2-Cell arrest[9]
	LSM14B	Infertility; Delayed onset of anaphase I and failure to enter MII after PBE (This study)
	MARF1	Infertility; GV-arrest[10]
	PATL2	Subfertility; Compromised oocyte maturation and poor developmental competence of oocytes and embryos[11]
	YBX2	Infertility; Mutant oocytes grow more slowly during the first wave of folliculogenesis, and aberrant spindle formation and chromosome congression during MI.[12]
	ZAR1	Infertility; Zygote arrest[13]
Cell cycle regulators	CKS2	Infertility; MI arrest[14]
	FBXO43	Infertility; Oocyte meiotic progression defects[15]
	MOS	Reduced fertility; Failure of mature eggs to arrest during meiosis, and spontaneous parthenogenetic activation of unfertilized eggs[16]
	WEE2	Precocious meiotic resumption (siRNA and a transgenic RNA interference in oocytes)[17]
ODPF and essential oogenic regulators	BMP15	Subfertility; Decreased ovulation and fertilization rates[18]
	FGF8	Cooperate with BMP15 to promote glycolysis in cumulus cells[19]
	GDF9	Infertility; A block in folliculogenesis at the primary follicle stage[20]
	GPR3	Subfertility; Precocious resumption of meiosis within antral follicles independently of an increase in luteinizing hormone[21]
	IL7	Functioning as an oocyte-secreted factor during meiotic reentry to integrate the function of the oocyte and cumulus cells[22]
	KIT	KIT-KIT Ligand interactions are important regulators of oogenesis and folliculogenesis.[23]
	OOSP1	Infertility; 2-Cell arrest or 4-Cell arrest[24]
	PDE3A	Infertility; GV arrest[25]
	RSPO2	Rspo2 loss-of-function ovaries lack secondary follicles.[26]
	ZPs	ZP1: Subfertility; Early embryonic loss). ZP2: Infertility; No 2-cell embryos are formed. ZP3: Infertility; No 2-cell embryos are formed.[27]
Epigenetic regulators	DICER1	Infertility; MI arrest[28]
	DNMTs	2-Cell arrest (*Dnmt1*-siRNA or *Dnmt3a*-siRNA), DNMT3L: Development of mouse embryos, produced from Dnmt3l$^{-/-}$females, are arrested by embryonic day 10.5.[29]

	DPPA3	Infertility; Excessive DNA methylation at the genome-wide level, including in the promoters of inactive genes[29]
	HELLS	Centromere instability and abnormal chromosome segregation[30]
	HIRA	A severe fertility phenotype; Defective development of follicles and embryo[31]
	NPM2	Subfertility or infertility; Failed preimplantation embryo development[32]
	TRIM28	Mater deletion leads to embryonic lethality due to misregulation of genomic imprinting.[33]
	UHRF1	Infertility; Increased incidence of aneuploidy and DNA damage, abnormal pre-implantation embryonic development[29]
Components of SCMC	NLRPs	NLRP4F: Decreased fecundity; Delayed preimplantation development[34]; NLRP5: Infertility; 2-Cell arrest[35]
	OOEP	Infertility; Pronuclear arrest[36]
	PADI6	Infertility; Dispersal of the cytoskeletal sheets[37]
	TLE6	Infertility; 2-Cell arrest[38]
	ZBED3	Reduced fecundity; Impaired and delayed development in a proportion of mutant embryos[39]
Transcriptional factors essential for oocyte development	BNC2	Infertility; Defects in oogenesis[40]
	BWRD1	Infertility; Pronuclear arrest[41]
	CTCF	Meiotic defects in oocytes[42]
	FOXO3	Infertility; Global follicular activation leading to oocyte death, early depletion of functional ovarian follicles[43]
	NOBOX	Infertility; Disruption of early folliculogenesis[44]

Table S3. Information of the antibodies used in this study

Antibodies	Source	Identifier
rabbit LSM14B	Bethyl	Cat# A305-851A-M
rabbit 4E-T/Eif4E-T	Bethyl	Cat# A300-706A-M
rabbit MASTL	Cell Signaling Technology	Cat#12069
rabbit Phospho-cdc2 (Tyr15)	Cell Signaling Technology	Cat#9111S
rabbit WEE1	Abcam	Cat# ab233540
rabbit MARF1	Proteintech	Custom-Made
rabbit LSM14A	Abcam	Cat# ab229277
rabbit SPIN1	Proteintech	Cat#12105-1-AP
rabbit MSY2	Abcam	Cat# ab154829
rabbit RBPMS2	Abcam	Cat# ab181098
goat BUBR1	Abcam	Cat# ab28193
rabbit CPEB1	Proteintech	Cat#13274-1-AP
rabbit DDX6	Proteintech	Cat#14632-1-AP
rabbit IGF2BP2	Proteintech	Cat#11601-1-AP
mouse GAPDH	Proteintech	Cat#60004-1-Ig
mouse BETA ACTIN	Proteintech	Cat#66009-1-Ig
mouse α-TUBULIN-FITC	Sigma	Cat# F216
human CENTROMERE	Antibodies Incorporated	Cat#15-234
mouse UHRF1	Santa Cruz	Cat# sc-373750
rabbit PATL2	Provided by Dr. Qing Sang and Lei Wang of Fudan University, Shanghai, China	
rabbit NLRP4F	Provided by Dr. Lei Li at Institute of Zoology, Chinese Academy of Sciences, Beijing, China	
IPKine HRP, Mouse anti-rabbit IgG LCS	Abbkine	Cat# A25022
Alexa Fluor-488 donkey anti- mouse IgG	Thermo Fisher Scientific	Cat# A21202
Alexa Fluor-488 donkey anti-goat IgG	Thermo Fisher Scientific	Cat# A11055
Alexa Fluor-594 donkey anti-rabbit IgG	Thermo Fisher Scientific	Cat# A21207
Alexa Fluor-594 donkey anti-human IgG	Jackson ImmunoResearch	Cat#709-585-149

Captions for Supplementary Videos and Datasets

Movie S1.
Time-lapse movie of meiotic maturation of a WT oocyte expressing β5-tubulin-EGFP (microtubules, green) and H2B-mCherry (chromosomes, red). Scale bar, 10 μm.

Movie S2.
Time-lapse movie of meiotic maturation of a *Lsm14b*-KO oocyte expressing β5-tubulin-EGFP (microtubules, green) and H2B-mCherry (chromosomes, red). Scale bar, 10 μm.

Movie S3.
Time-lapse movie of the recovery of the LSM14B-mKate (in red) formed P-body-like large fluorescent puncta in a live HEK293 cell. Scale bar, 5 μm.

Movie S4.
Time-lapse movie of the recovery of the LSM14B-mKate (in red) formed P-body-like large fluorescent puncta in a live early-stage growing mouse oocyte. Scale bar, 10 μm.

Dataset S1.
RBPome dataset of mouse GV-stage fully-grown oocytes. Two independent *mRNA* interactome capture (RIC) experiments were carried out with each using 25000 oocytes.

Dataset S2.
List of significantly changed transcripts in *Lsm14b*-KO oocytes identified by RNA-seq analysis (KO *vs* WT, $P<0.05$)

Dataset S3.
List of significantly changed proteins in *Lsm14b*-KO oocytes identified by proteomics analysis (KO *vs* WT, $P<0.05$)

Dataset S4.
List of LSM14B-bound mRNAs identified by CLIP and LACE-Seq analyses.

Dataset S5.
List of the mRNAs with translation efficiency significantly changed in *Lsm14b*-KO oocytes.

Dataset S6.
List of the LSM14B-interacting proteins identified by IP-MS. Two independent *IP-MS* experiments were carried out with each using 8000 oocytes.

Dataset S7.
List of the overlapped proteins in both the LSM14B IP-MS dataset and the P-body protein dataset.

Supplementary References

[1] A. Hubstenberger, M. Courel, M. Benard, S. Souquere, M. Ernoult-Lange, R. Chouaib, Z. Yi, J. B. Morlot, A. Munier, M. Fradet, M. Daunesse, E. Bertrand, G. Pierron, J. Mozziconacci, M. Kress, D. Weil, *Mol Cell* **2017**, *68* (1), 144, https://doi.org/10.1016/j.molcel.2017.09.003.

[2] C. Yu, S. Y. Ji, Q. Q. Sha, Y. Dang, J. J. Zhou, Y. L. Zhang, Y. Liu, Z. W. Wang, B. Hu, Q. Y. Sun, S. C. Sun, F. Tang, H. Y. Fan, *Nat Struct Mol Biol* **2016**, *23* (5), 387, https://doi.org/10.1038/nsmb.3204.

[3] Q. Q. Sha, J. L. Yu, J. X. Guo, X. X. Dai, J. C. Jiang, Y. L. Zhang, C. Yu, S. Y. Ji, Y. Jiang, S. Y. Zhang, L. Shen, X. H. Ou, H. Y. Fan, *EMBO J* **2018**, *37* (24), https://doi.org/10.15252/embj.201899333.

[4] W. J. Racki, J. D. Richter, *Development* **2006**, *133* (22), 4527, https://doi.org/10.1242/dev.02651.

[5] J. Tay, J. D. Richter, *Dev Cell* **2001**, *1* (2), 201, https://doi.org/10.1016/s1534-5807(01)00025-9.

[6] J. Chen, C. Melton, N. Suh, J. S. Oh, K. Horner, F. Xie, C. Sette, R. Blelloch, M. Conti, *Genes Dev* **2011**, *25* (7), 755, https://doi.org/25/7/755 [pii] 10.1101/gad.2028911.

[7] Y. Kato, T. Iwamori, Y. Ninomiya, T. Kohda, J. Miyashita, M. Sato, Y. Saga, *EMBO Rep* **2019**, *20* (12), e48251, https://doi.org/10.15252/embr.201948251.

[8] L. Yu, H. Zhang, X. Guan, D. Qin, J. Zhou, X. Wu, *Development* **2021**, *148* (2), https://doi.org/10.1242/dev.196931.

[9] H. B. Liu, T. Muhammad, Y. Guo, M. J. Li, Q. Q. Sha, C. X. Zhang, H. Liu, S. G. Zhao, H. Zhao, H. Zhang, Y. Z. Du, K. Sun, K. Liu, G. Lu, X. J. Guo, J. Sha, H. Y. Fan, F. Gao, Z. J. Chen, *Adv Sci (Weinh)* **2019**, *6* (15), 1900295, https://doi.org/10.1002/advs.201900295.

[10] Y. Q. Su, K. Sugiura, F. Sun, J. K. Pendola, G. A. Cox, M. A. Handel, J. C. Schimenti, J. J. Eppig, *Science* **2012**, *335* (6075), 1496, https://doi.org/10.1126/science.1214680.

[11] M. Christou-Kent, Z. E. Kherraf, A. Amiri-Yekta, E. Le Blevec, T. Karaouzene, B. Conne, J. Escoffier, S. Assou, A. Guttin, E. Lambert, G. Martinez, M. Boguenet, S. Fourati Ben Mustapha, I. Cedrin Durnerin, L. Halouani, O. Marrakchi, M. Makni, H. Latrous, M. Kharouf, C. Coutton, N. Thierry-Mieg, S. Nef, S. P. Bottari, R. Zouari, J. P. Issartel, P. F. Ray, C. Arnoult, *EMBO Mol Med* **2018**, *10* (5), https://doi.org/10.15252/emmm.201708515.

[12] S. Medvedev, H. Pan, R. M. Schultz, *Biol Reprod* **2011**, *85* (3), 575, https://doi.org/10.1095/biolreprod.111.091710.

[13] X. Wu, M. M. Viveiros, J. J. Eppig, Y. Bai, S. L. Fitzpatrick, M. M. Matzuk, *Nat Genet* **2003**, *33* (2), 187, https://doi.org/10.1038/ng1079.

[14] C. H. Spruck, M. P. de Miguel, A. P. Smith, A. Ryan, P. Stein, R. M. Schultz, A. J. Lincoln, P. J. Donovan, S. I. Reed, *Science* **2003**, *300* (5619), 647, https://doi.org/10.1126/science.1084149.

[15] L. Gopinathan, R. Szmyd, D. Low, M. K. Diril, H. Y. Chang, V. Coppola, K. Liu, L. Tessarollo, E. Guccione, A. M. M. van Pelt, P. Kaldis, *Cell Rep* **2017**, *20* (3), 697, https://doi.org/10.1016/j.celrep.2017.06.033.

[16] a) W. H. Colledge, M. B. Carlton, G. B. Udy, M. J. Evans, *Nature* **1994**, *370* (6484), 65, https://doi.org/10.1038/370065a0; b) N. Hashimoto, N. Watanabe, Y. Furuta, H. Tamemoto, N. Sagata, M. Yokoyama, K. Okazaki, M. Nagayoshi, N. Takeda, Y. Ikawa, et al., *Nature* **1994**, *370* (6484), 68, https://doi.org/10.1038/370068a0.

[17] S. J. Han, R. Chen, M. P. Paronetto, M. Conti, *Curr Biol* **2005**, *15* (18), 1670, https://doi.org/10.1016/j.cub.2005.07.056.

[18] C. Yan, P. Wang, J. DeMayo, F. J. DeMayo, J. A. Elvin, C. Carino, S. V. Prasad, S. S. Skinner, B. S. Dunbar, J. L. Dube, A. J. Celeste, M. M. Matzuk, *Mol Endocrinol* **2001**, *15* (6), 854, https://doi.org/10.1210/mend.15.6.0662.

[19] K. Sugiura, Y. Q. Su, F. J. Diaz, S. A. Pangas, S. Sharma, K. Wigglesworth, M. J. O'Brien, M. M. Matzuk, S. Shimasaki, J. J. Eppig, *Development* **2007**, *134* (14), 2593, https://doi.org/10.1242/dev.006882.

[20] J. Dong, D. F. Albertini, K. Nishimori, T. R. Kumar, N. Lu, M. M. Matzuk, *Nature* **1996**, *383* (6600), 531, https://doi.org/10.1038/383531a0.

[21] L. M. Mehlmann, Y. Saeki, S. Tanaka, T. J. Brennan, A. V. Evsikov, F. L. Pendola, B. B. Knowles, J. J. Eppig, L. A. Jaffe, *Science* **2004**, *306* (5703), 1947, https://doi.org/306/5703/1947 [pii]10.1126/science.1103974.

[22] H. Cakmak, F. Franciosi, A. M. Zamah, M. I. Cedars, M. Conti, *Proc Natl Acad Sci U S A* **2016**, *113* (9), 2424, https://doi.org/10.1073/pnas.1519990113.

[23] M. A. Driancourt, K. Reynaud, R. Cortvrindt, J. Smitz, *Rev Reprod* **2000**, *5* (3), 143, https://doi.org/10.1530/ror.0.0050143.

[24] F. Tashiro, M. Kanai-Azuma, S. Miyazaki, M. Kato, T. Tanaka, S. Toyoda, E. Yamato, H. Kawakami, T. Miyazaki, J. Miyazaki, *Genes Cells* **2010**, *15* (8), 813, https://doi.org/10.1111/j.1365-2443.2010.01420.x.

[25] S. Masciarelli, K. Horner, C. Liu, S. H. Park, M. Hinckley, S. Hockman, T. Nedachi, C. Jin, M. Conti, V. Manganiello, *J Clin Invest* **2004**, *114* (2), 196, https://doi.org/10.1172/JCI21804.

[26] M. C. De Cian, E. P. Gregoire, M. Le Rolle, S. Lachambre, M. Mondin, S. Bell, C. J. Guigon, A. A. Chassot, M. C. Chaboissier, *Cell Death Differ* **2020**, *27* (10), 2856, https://doi.org/10.1038/s41418-020-0547-7.

[27] P. M. Wassarman, E. S. Litscher, *Elife* **2022**, *11*, https://doi.org/10.7554/eLife.76106.

[28] a) E. P. Murchison, P. Stein, Z. Xuan, H. Pan, M. Q. Zhang, R. M. Schultz, G. J. Hannon, *Genes Dev* **2007**, *21* (6), 682, https://doi.org/10.1101/gad.1521307; b) F. Tang, M. Kaneda, D. O'Carroll, P. Hajkova, S. C. Barton, Y. A. Sun, C. Lee, A. Tarakhovsky, K. Lao, M. A. Surani, *Genes Dev* **2007**, *21* (6), 644, https://doi.org/21/6/644 [pii] 10.1101/gad.418707.

[29] Y. Li, Z. Zhang, J. Chen, W. Liu, W. Lai, B. Liu, X. Li, L. Liu, S. Xu, Q. Dong, M. Wang, X. Duan, J. Tan, Y. Zheng, P. Zhang, G. Fan, J. Wong, G. L. Xu, Z. Wang, H. Wang, S. Gao, B. Zhu, *Nature* **2018**, *564* (7734), 136, https://doi.org/10.1038/s41586-018-0751-5.

[30] C. Baumann, W. Ma, X. Wang, M. K. Kandasamy, M. M. Viveiros, R. De La Fuente, *Nat Commun* **2020**, *11* (1), 4486, https://doi.org/10.1038/s41467-020-18009-3.

[31] a) B. Nashun, P. W. Hill, S. A. Smallwood, G. Dharmalingam, R. Amouroux, S. J. Clark, V. Sharma, E. Ndjetehe, P. Pelczar, R. J. Festenstein, G. Kelsey, P. Hajkova, *Mol Cell* **2015**, *60* (4), 611, https://doi.org/10.1016/j.molcel.2015.10.010; b) R. Smith, A. Susor, H. Ming, J. Tait, M. Conti, Z. Jiang, C. J. Lin, *Development* **2022**, *149* (5), https://doi.org/10.1242/dev.200044.

[32] K. H. Burns, M. M. Viveiros, Y. Ren, P. Wang, F. J. DeMayo, D. E. Frail, J. J. Eppig, M. M. Matzuk, *Science* **2003**, *300* (5619), 633, https://doi.org/10.1126/science.1081813.

[33] D. M. Messerschmidt, W. de Vries, M. Ito, D. Solter, A. Ferguson-Smith, B. B. Knowles, *Science* **2012**, *335* (6075), 1499, https://doi.org/10.1126/science.1216154.

[34] D. Qin, Z. Gao, Y. Xiao, X. Zhang, H. Ma, X. Yu, X. Nie, N. Fan, X. Wang, Y. Ouyang, Q. Y. Sun, Z. Yi, L. Li, *Development* **2019**, *146* (20), https://doi.org/10.1242/dev.183616.

[35] Z. B. Tong, L. Gold, K. E. Pfeifer, H. Dorward, E. Lee, C. A. Bondy, J. Dean, L. M. Nelson, *Nat Genet* **2000**, *26* (3), 267, https://doi.org/10.1038/81547.

[36] L. Li, B. Baibakov, J. Dean, *Dev Cell* **2008**, *15* (3), 416, https://doi.org/10.1016/j.devcel.2008.07.010.

[37] G. Esposito, A. M. Vitale, F. P. Leijten, A. M. Strik, A. M. Koonen-Reemst, P. Yurttas, T. J. Robben, S. Coonrod, J. A. Gossen, *Mol Cell Endocrinol* **2007**, *273* (1-2), 25, https://doi.org/10.1016/j.mce.2007.05.005.

[38] X. J. Yu, Z. Yi, Z. Gao, D. Qin, Y. Zhai, X. Chen, Y. Ou-Yang, Z. B. Wang, P. Zheng, M. S. Zhu, H. Wang, Q. Y. Sun, J. Dean, L. Li, *Nat Commun* **2014**, *5*, 4887, https://doi.org/10.1038/ncomms5887.

[39] Z. Gao, X. Zhang, X. Yu, D. Qin, Y. Xiao, Y. Yu, Y. Xiang, X. Nie, X. Lu, W. Liu, Z. Yi, L. Li, *J Mol Cell Biol* **2018**, *10* (1), 74, https://doi.org/10.1093/jmcb/mjx035.

[40] A. Vanhoutteghem, B. Delhomme, F. Herve, I. Nondier, J. M. Petit, M. Araki, K. Araki, P. Djian, *Mech Dev* **2016**, *140*, 53, https://doi.org/10.1016/j.mod.2016.02.002.

[41] D. L. Philipps, K. Wigglesworth, S. A. Hartford, F. Sun, S. Pattabiraman, K. Schimenti, M. Handel, J. J. Eppig, J. C. Schimenti, *Dev Biol* **2008**, *317* (1), 72, https://doi.org/10.1016/j.ydbio.2008.02.018.

[42] A. M. Fedoriw, P. Stein, P. Svoboda, R. M. Schultz, M. S. Bartolomei, *Science* **2004**, *303* (5655), 238, https://doi.org/10.1126/science.1090934.

[43] D. H. Castrillon, L. Miao, R. Kollipara, J. W. Horner, R. A. DePinho, *Science* **2003**, *301* (5630), 215, https://doi.org/10.1126/science.1086336.

[44] A. Rajkovic, S. A. Pangas, D. Ballow, N. Suzumori, M. M. Matzuk, *Science* **2004**, *305* (5687), 1157, https://doi.org/10.1126/science.1099755.
